# Meta-Analysis of Maternal and Fetal Transcriptomic Data Elucidates the Role of Adaptive and Innate Immunity in Preterm Birth

**DOI:** 10.3389/fimmu.2018.00993

**Published:** 2018-05-09

**Authors:** Bianca Vora, Aolin Wang, Idit Kosti, Hongtai Huang, Ishan Paranjpe, Tracey J. Woodruff, Tippi MacKenzie, Marina Sirota

**Affiliations:** ^1^Institute for Computational Health Sciences, University of California San Francisco, San Francisco, CA, United States; ^2^Department of Bioengineering and Therapeutic Sciences, University of California San Francisco, San Francisco, CA, United States; ^3^Program on Reproductive Health and the Environment, Department of Obstetrics, Gynecology and Reproductive Sciences, University of California San Francisco, San Francisco, CA, United States; ^4^Department of Pediatrics, University of California San Francisco, San Francisco, CA, United States; ^5^Center for Maternal-Fetal Precision Medicine, University of California San Francisco, San Francisco, CA, United States; ^6^Department of Surgery, University of California San Francisco, San Francisco, CA, United States

**Keywords:** preterm birth, meta-analysis, transcriptomics, immunology, pregnancy

## Abstract

Preterm birth (PTB) is the leading cause of newborn deaths around the world. Spontaneous preterm birth (sPTB) accounts for two-thirds of all PTBs; however, there remains an unmet need of detecting and preventing sPTB. Although the dysregulation of the immune system has been implicated in various studies, small sizes and irreproducibility of results have limited identification of its role. Here, we present a cross-study meta-analysis to evaluate genome-wide differential gene expression signals in sPTB. A comprehensive search of the NIH genomic database for studies related to sPTB with maternal whole blood samples resulted in data from three separate studies consisting of 339 samples. After aggregating and normalizing these transcriptomic datasets and performing a meta-analysis, we identified 210 genes that were differentially expressed in sPTB relative to term birth. These genes were enriched in immune-related pathways, showing upregulation of innate immunity and downregulation of adaptive immunity in women who delivered preterm. An additional analysis found several of these differentially expressed at mid-gestation, suggesting their potential to be clinically relevant biomarkers. Furthermore, a complementary analysis identified 473 genes differentially expressed in preterm cord blood samples. However, these genes demonstrated downregulation of the innate immune system, a stark contrast to findings using maternal blood samples. These immune-related findings were further confirmed by cell deconvolution as well as upstream transcription and cytokine regulation analyses. Overall, this study identified a strong immune signature related to sPTB as well as several potential biomarkers that could be translated to clinical use.

## Introduction

Preterm birth (PTB), which is defined as giving birth before completion of 37 weeks of gestation, is the leading cause of newborn deaths worldwide. In 2010, 14.9 million babies were born preterm, accounting for 11.1% of all births across 184 countries, with the highest PTB rates occurring in Africa and North America (1). This high incidence of PTB is concerning since 29% of all neonatal deaths worldwide, approximately 1 million deaths total, are accounted to complications in PTB (2). Furthermore, children born prematurely are at increased risk for a milieu of short- and long-term complications including motor, cognitive, and behavioral impairments (3, 4).

Approximately 30% of PTBs are medically indicated due to maternal or fetal conditions; the other two-thirds are categorized as spontaneous preterm births (sPTB) that include spontaneous preterm labor and preterm premature rupture of the membranes (5). PTB is a syndrome with multiple etiologies. Numerous signs point to genetic factors as playing a role in birth timing including the observations that PTBs are likely to recur in mothers, women who are born preterm are more likely to deliver prematurely, and sisters of women who have delivered prematurely are at an increased risk of delivering preterm. Furthermore, twin studies suggest that genetics account for approximately one-third of the variation in PTB (6, 7). Other factors shown to influence risk for PTB include those associated with adverse lifestyle and behavior, such as stress, smoking, drug use, and nutrition (8). Although a variety of social (9, 10), environmental, and maternal factors have been implicated in PTB, causes of sPTB have remained largely mysterious and therefore, in most instances, not amenable to effective interventions. Thus far, there exists no universal detection method to predict sPTB or intervention approach to prolong labor and extend the pregnancy to term. The complexity and multiple etiologies of sPTB, along with the inconsistency in clinical phenotyping and non-uniform classification system, have limited the identification of genetic factors and clinically relevant biomarkers (11).

Over the years, many different mechanisms have been identified to be associated with sPTB, including breakdown of maternal–fetal tolerance, decidual senescence, uterine overdistension, and procoagulant activity (12, 13). One particularly interesting mechanism that has been implicated is the dysregulation of the interplay between the maternal innate and adaptive immune systems. The innate immune system, also known as the non-specific immune system, comprises cells and mechanisms including but not limited to macrophages, toll-like receptors, neutrophils, and cytokines which aid in host defense from infection (14, 15). This sub-system is responsible for the generalized, non-specific immune response, inflammation, and activation of the adaptive immune system through antigen presentation (14, 15). Contrastingly, the adaptive immune system comprises lymphocytes, specifically T cells and B cells, which are specialized white blood cells that provide long-term immunity (14, 15). In pregnancy, regulatory T-cells proliferate after implantation and function to prevent rejection of the fetus by creating an anti-inflammatory environment (16, 17). However, for labor to initiate and progress, the maternal immune system switches to a pro-inflammatory state by activating the pro-inflammatory nuclear factor-kB signaling pathway, which leads to an increase in the production of cytokines, chemokines, and interleukins and allows for infiltration of the fetal/maternal interface by activating leukocytes (16–19). The location and function of each immune cell is critical to sustain pregnancy to term; it has been proposed that a premature shift from the anti-inflammatory to the pro-inflammatory state, and therefore a disruption in the balance of innate and adaptive immunity, could result in preterm labor and delivery (19).

There is a need to understand the mechanisms by which preterm labor is affected which could then lead to identification, intervention, and prevention. Identifying immune-related genetic signatures as well as clinically relevant diagnostic biomarkers specific to sPTB would enhance our ability to discern women who are at an elevated risk for delivering prematurely. However, findings have been limited due to small sample size and issues with irreproducibility (20). Meta-analysis, which combines information from multiple existing studies, is a powerful tool that improves reliability, generalizability, and ability to detect differential gene expression by larger statistical power (20). With the development of databases such as the National Institute of Health Gene Expression Omnibus (NIH GEO) and Array Express, gene expression meta-analysis has been applied to investigate different disease subtypes and discover novel biomarkers (21–24). In the area of obstetrics, a recent study performed a meta-analysis which integrated diverse types of genomic data, overlaying evolutionary data, and placental expression data in an effort to elucidate genes that may be involved in parturition and disrupt pregnancy (25).

As discussed in a recent systematic review (26), although there have been 134 genome-wide transcriptomic studies related to pregnancy and PTB, most of these studies have focused on PTB related to preeclampsia (one of the medical indications of PTB). sPTB was investigated in only 7% of all studies and 18% of preterm studies, even though sPTB is responsible for over two-thirds of all PTBs. Furthermore, 61% of the studies focused on placental tissue, which has limited utility in the diagnostic setting and upon comparison of results from the different studies, there was very limited overlap among differentially expressed genes; only 2 genes of 6,444 differentially expressed genes identified were present in 10 or more gene expression studies (26). Therefore, there exists a need to aggregate data and perform meta-analyses to elucidate gene signatures that are robust and can be reproduced in studies of maternal blood, which allows for discovery of biomarkers that can be implemented as part of the standard prenatal care. The NIH GEO database has three sPTB related, publicly available datasets which have all been analyzed separately before. The first study, which included women who were diagnosed with threatened preterm labor (median gestational age: 32 weeks), found 469 differentially expressed genes and significantly increased leukocyte and neutrophil counts in women who had sPTB within 48 h after initiation of labor (27). The second study, also by Heng et al., collected samples at two different time points and found no differentially expressed genes in the second trimester and 26 differentially expressed genes in the third trimester when comparing sPTB and term birth (28). The last study analyzed eight tissue types, comparing women who delivered preterm and term with or without labor; they found that pregnancy was maintained by downregulation of chemokines at the maternal–fetal interface but the work has not been published.

Using these three datasets, we performed a cross-study meta-analysis which identified a set of significant differentially expressed genes in maternal blood, many of which were immune related and a few of which could translate to clinically relevant biomarkers. An additional analysis of measurements collected during mid-gestation in one study revealed a smaller set of significant genes that were differentially expressed over time. Finally, a complementary analysis of fetal cord blood (CB) revealed that there were a number of differentially expressed genes on the fetal side, many of which overlapped with the significant genes in maternal blood and showed opposing changes in regulation.

## Results

### Datasets

We identified three datasets, from the National Center for Biotechnology Information (NCBI) Gene Expression Omnibus (GEO) database (23, 24), which were comprised of whole blood gene expression profiles from women who delivered preterm and term, respectively. These three studies (GSE46510, GSE59491, and GSE73685) included 339 maternal whole blood samples, 134 from women who delivered preterm, and 205 from those delivered at term. The gestational age of the preterm deliveries ranged from 24.4 to 36.9 weeks with a median of 34 weeks. One study (GSE59491) collected blood samples at two different time points, second trimester (17–23 weeks) and third trimester (27–33 weeks), respectively. In addition to whole blood samples, another study used in the meta-analysis (GSE73685) collected RNA samples from seven other different types of tissues including amnion, CB, chorion, decidua, fundus, lower segment, and placenta (Table 1).

**Table 1:** Datasets used in discovery analyses.

Dataset	Year	Author	Platform	Sample types	Preterm births	Term births	Gestational age at sampling*
GSE46510	2014	Heng	GPL16311	Maternal whole blood	75	79	32 (24–36)
GSE59491	2016	Heng	GPL18964	Maternal whole blood	51 (T2) 47 (T3)	114 (T2) 114 (T3)	19 (17–20) 29 (27–33)
GSE73685	2016	Baldwin	GPL6244	Amnion (A)	12	12	NR
				Cord blood	11	12	
				Chorion (C)	12	12	
				Decidua (D)	11	12	
				Fundus (F)	10	10	
				Lower segment	12	12	
				Placenta (P)	12	9	
				Maternal whole blood (WB)	12	12	

*Median (range) reported*.

*NR, not reported; T2, second trimester; T3, third trimester*.

### Overview

**Figure 1:**
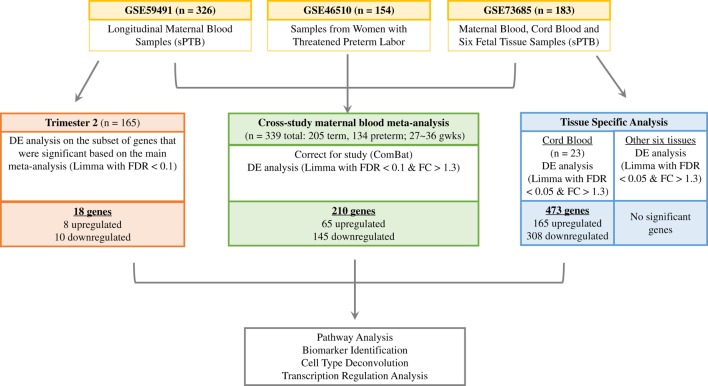
Analysis of relationship of gene expression differences in term vs. preterm birth. We identified three independent studies from the Gene Expression Omnibus database (in yellow) to perform a meta-analysis using third trimester maternal blood samples (in green), an additional differential expression analysis with second trimester samples from GSE59491 (in orange), and a tissue-specific analysis with samples from GSE73685 (in blue).

Our primary goal was to perform a meta-analysis to identify potential maternal plasma biomarkers by evaluating differentially expressed genes associated with sPTB and investigating whether certain cell types are enriched in sPTB, using time-matched maternal data from the three independent studies. Taking advantage of the repeated samples collected in mid-gestation from study GSE59491 and samples collected from seven additional tissues in study GSE73685, we performed secondary analyses to identify potential common gene expression signatures across different gestational stages and different tissues and to investigate the potential maternal–fetal interplay at the transcriptomic level. We investigated and compared the transcriptomic signature that was identified as part of the maternal meta-analysis to what was observed earlier in the pregnancy. The second additional analysis investigated differential gene expression in various tissue types to identify tissue specific transcriptomic signatures (Figure 1). Each of the signatures was further interrogated through pathway and transcriptional regulation analysis.

### Cross-Study Gene Expression Meta-Analysis in Maternal Blood

Samples from the three studies were pooled together based on gestational age at time of sample collection. The women were split into two groups based only on whether they delivered before or after 37 weeks of gestation, with no regard to time of delivery relative to the initiation of labor.

**Figure 2:**
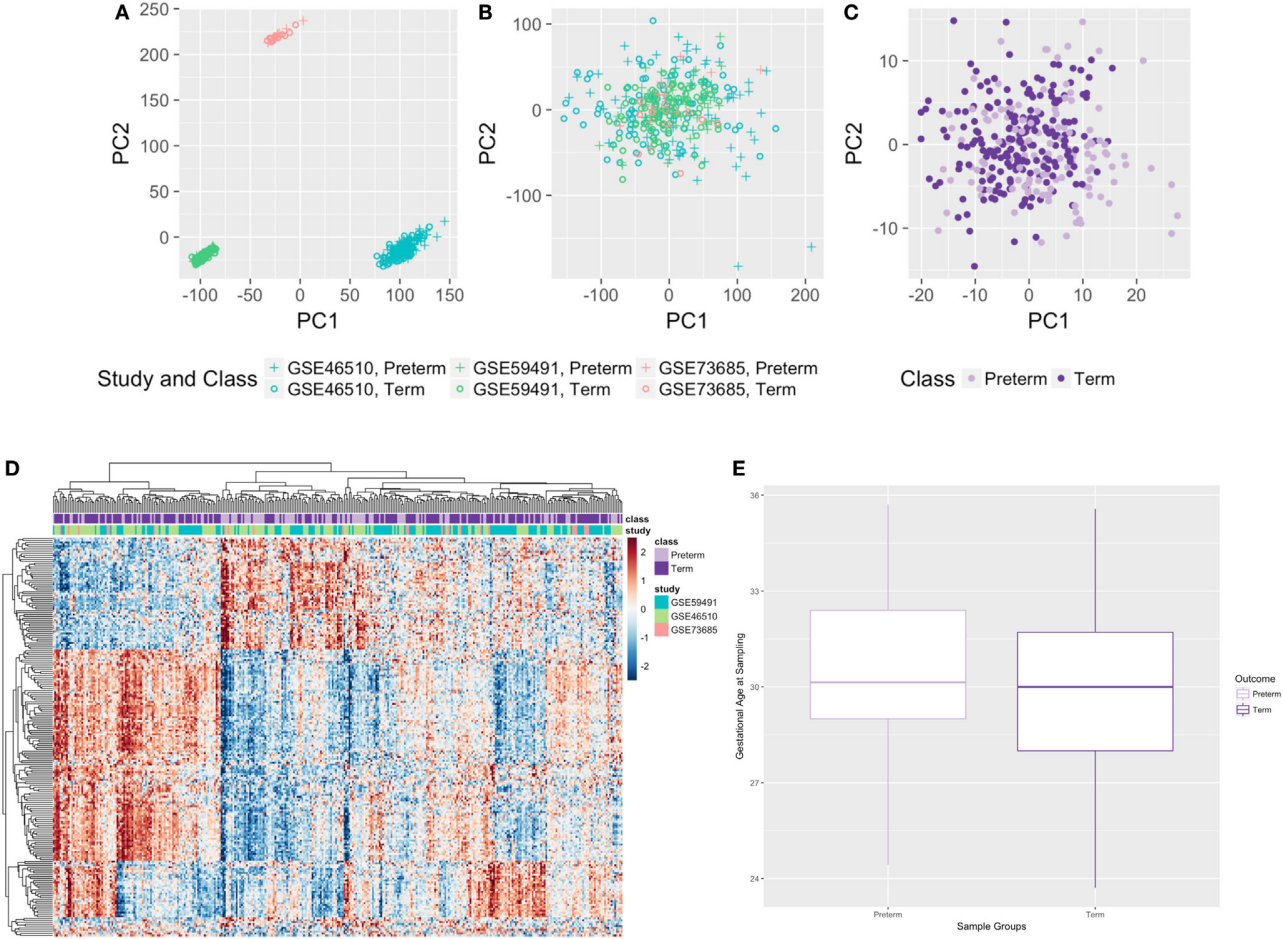
Results from the cross-study meta-analysis and distribution of gestational age at sampling. **(A,B)** Principal component analysis plots with all genes before **(A)** and after **(B)** ComBat. **(C,D)** Principal component analysis plot **(C)** and heatmap **(D)** of all samples based on 210 significant differentially expressed genes. **(E)** Gestational age at sampling was not significantly different between preterm and term maternal whole blood samples (*n* = 315, *p*-value = 0.125).

When pooling samples from the different studies, study-specific differences in gene expression were seen (Figure 2A) and corrected for using ComBat (29) to eliminate such biases (Figure 2B). When we imposed a false discovery rate (FDR) of 0.1, the normalized, merged gene dataset of 17,337 was reduced to 4,648 significant genes. Setting a significance threshold at a fold change (FC) of 1.3-fold increase or decrease in gene expression (30) for PTB samples, relative to term birth, condensed our gene list from 4,648 genes to 210 differentially expressed genes (FC range: 0.46–1.94) (Figure 2C), with 65 genes upregulated and 145 genes downregulated (Table S1 in Supplementary Material). We saw clustering of preterm samples and term samples based on the 210 significant genes; however, we did not see any clustering by study (Figure 2D). Only third trimester samples from GSE59491 were used in the meta-analysis to better time-match all samples (Figure 2E).

**Figure 3:**
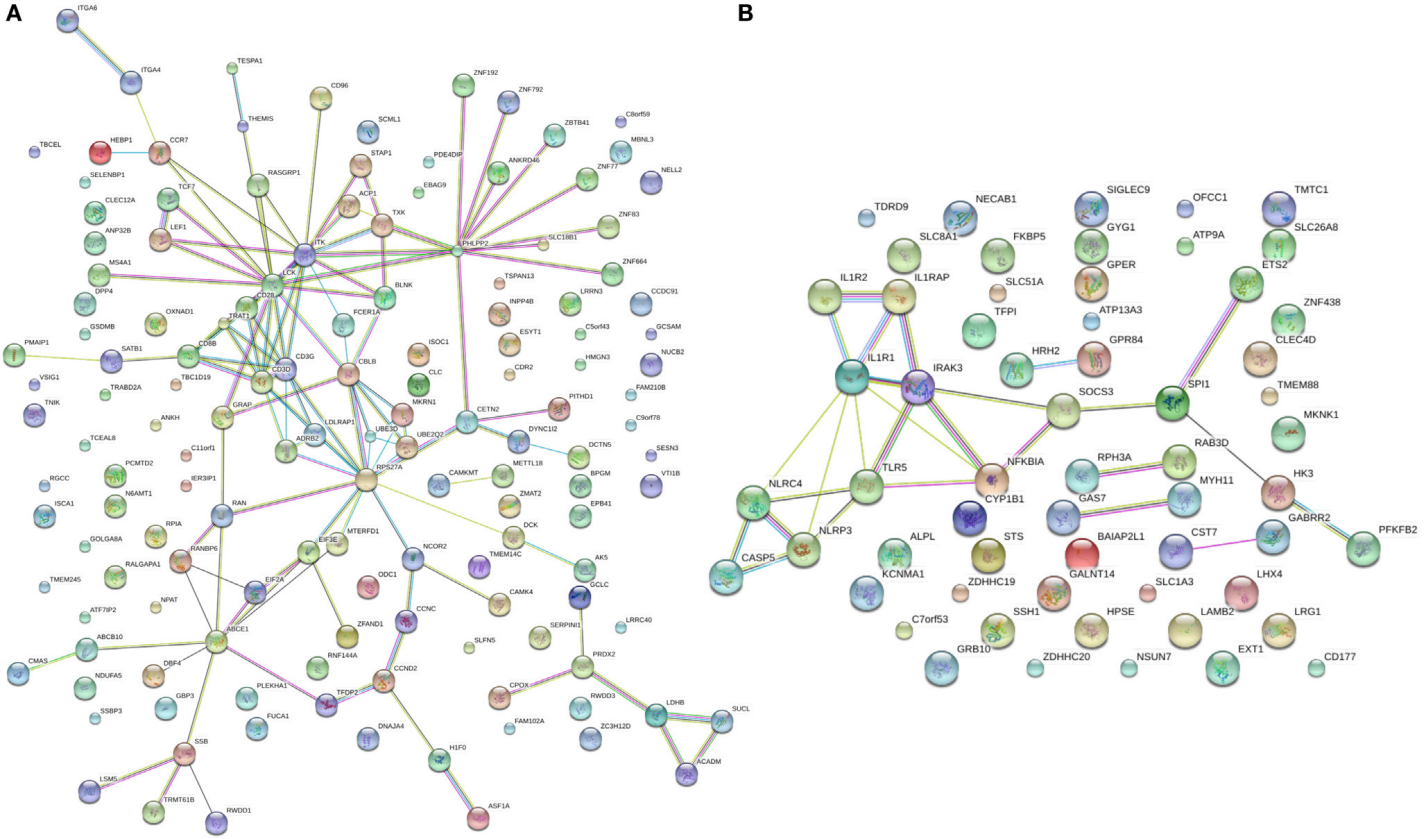
STRING connectivity networks based on 210 differentially expressed genes. **(A,B)** Connectivity networks for significantly downregulated **(A)** and upregulated **(B)** genes from meta-analysis.

**Figure 4:**
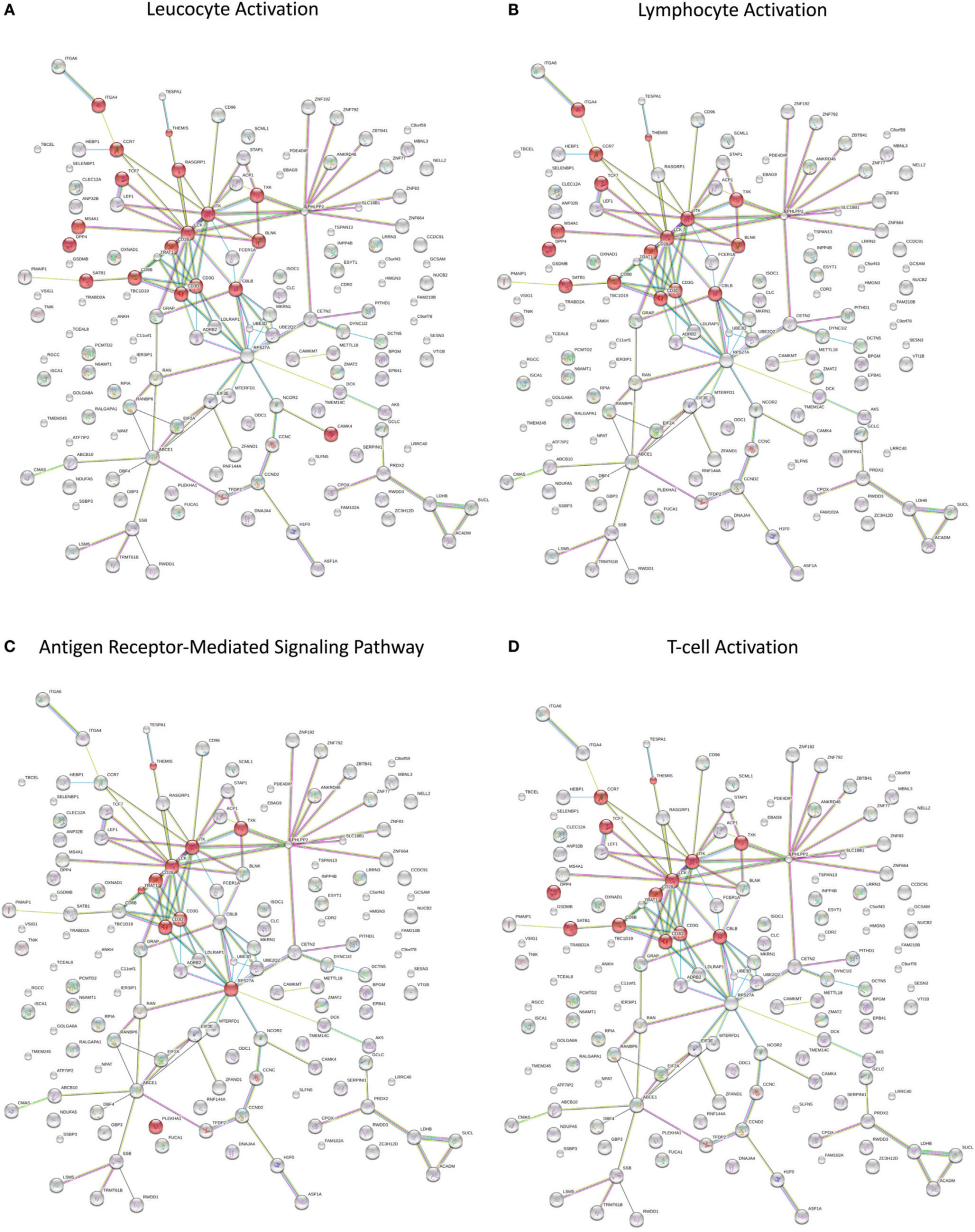
**(A–D)** Network visualization of functionally enriched GO biological processes in significantly downregulated genes from the meta-analysis.

Splitting our list of 210 genes into two sub-groups based on whether they were upregulated or downregulated in PTB, we found that the downregulated genes demonstrated strong network connectivity using the STRING database (31–33) (Figure 3A) and were functionally enriched in 36 different pathways using the ToppFun database (34) (Table 2), more than half of which were immune related. Specifically, the downregulated genes were highly involved in the adaptive immune response, showing significant clustering and connectivity in Gene Ontology Consortium (GO) biological processes (Table S2 in Supplementary Material) including antigen receptor-mediated signaling pathway, leukocyte activation, lymphocyte activation, and T-cell activation (Figures 4A–D). Furthermore, there were six genes (*CD8B, CLC, DPP4, NELL2, SERPINI1*, and *NUCB2*) of 145 downregulated genes that were found to be secreted as proteins in humans from the UnitProt database (35) (Table 3). The 65 upregulated genes showed less network connectivity relative to the downregulated genes (Figure 3B). Although the majority (5 of 6) of the functionally enriched pathways were immune related (Table 2), the upregulated genes were specifically involved in the innate immune response, a stark contrast to the downregulated genes. In addition, there were 9 genes from the 65 upregulated genes that were found be secreted as proteins in humans [IL-1 receptor type I (*IL-1R1*), *IL-1R2, IL-1RAP, HPSE, NLRP3*, tissue factor pathway inhibitor (*TFPI*), *LRG1, CST7, LAMB2*] in the UniProt database (35) (Table 3).

**Table 2:** Functionally enriched pathways from cross-study meta-analysis.

ID	Name	Source	*p*-Value	FDR B&H	Genes from input	Genes in annotation
**Upregulated**						
M12095	Signal transduction through IL1R*	MSigDB C2 BIOCARTA (v5.1)	6.17E−06	3.04E−03	4	33
1269320	Interleukin-1 signaling*	BioSystems: REACTOME	2.38E−05	5.86E−03	4	46
1457780	Neutrophil degranulation*	BioSystems: REACTOME	6.52E−05	1.07E−02	9	492
1269203	Innate Immune System*	BioSystems: REACTOME	1.81E−04	2.23E−02	14	1312
137944	*IL1*-mediated signaling events*	BioSystems: Pathway Interaction Database	2.84E−04	2.54E−02	3	35
82974	Starch and sucrose metabolism	BioSystems: KEGG	3.10E−04	2.54E−02	3	36
**Downregulated**						
M1467	The Co-Stimulatory Signal During T-cell Activation*	MSigDB C2 BIOCARTA (v5.1)	3.46E−07	3.21E−04	5	21
83080	T cell receptor signaling pathway*	BioSystems: KEGG	8.04E−07	3.66E−04	8	103
138055	TCR signaling in naive CD8+ T cells*	BioSystems: Pathway Interaction Database	1.24E−06	3.66E−04	6	48
1269171	Adaptive Immune System*	BioSystems: REACTOME	1.58E−06	3.66E−04	20	826
137998	TCR signaling in naive CD4+ T cells*	BioSystems: Pathway Interaction Database	4.71E−06	8.73E−04	6	60
1269175	Generation of second messenger molecules	BioSystems: REACTOME	5.89E−06	9.10E−04	5	36
1269174	Translocation of ZAP-70 to Immunological synapse*	BioSystems: REACTOME	1.77E−05	2.10E−03	4	22
M9526	T Cell Signal Transduction*	MSigDB C2 BIOCARTA (v5.1)	1.81E−05	2.10E−03	5	45
1269173	Phosphorylation of CD3 and TCR zeta chains*	BioSystems: REACTOME	3.00E−05	2.98E−03	4	25
1269172	TCR signaling*	BioSystems: REACTOME	3.32E−05	2.98E−03	7	124
1269182	PD-1 signaling*	BioSystems: REACTOME	3.53E−05	2.98E−03	4	26
M16519	HIV Induced T Cell Apoptosis*	MSigDB C2 BIOCARTA (v5.1)	5.98E−05	4.62E−03	3	11
83078	Hematopoietic cell lineage*	BioSystems: KEGG	7.48E−05	5.34E−03	6	97
M10765	Lck and Fyn tyrosine kinases in initiation of TCR Activation*	MSigDB C2 BIOCARTA (v5.1)	1.03E−04	6.46E−03	3	13
1269176	Downstream TCR signaling*	BioSystems: REACTOME	1.05E−04	6.46E−03	6	103
M13247	T Cytotoxic Cell Surface Molecules*	MSigDB C2 BIOCARTA (v5.1)	1.30E−04	7.07E−03	3	14
M6427	T Helper Cell Surface Molecules*	MSigDB C2 BIOCARTA (v5.1)	1.30E−04	7.07E−03	3	14
83125	Primary immunodeficiency*	BioSystems: KEGG	1.47E−04	7.55E−03	4	37
1269177	Costimulation by the CD28 family*	BioSystems: REACTOME	1.90E−04	9.25E−03	5	73
169352	Regulation of Wnt-mediated beta catenin signaling and target gene transcription	BioSystems: Pathway Interaction Database	2.75E−04	1.27E−02	5	79
1269183	Signaling by the B Cell Receptor (BCR)*	BioSystems: REACTOME	3.25E−04	1.42E−02	8	236
M16966	Stathmin and breast cancer resistance to antimicrotubule agents	MSigDB C2 BIOCARTA (v5.1)	3.36E−04	1.42E−02	3	19
M18215	Role of Tob in T-cell activation*	MSigDB C2 BIOCARTA (v5.1)	4.57E−04	1.83E−02	3	21
1269201	Immunoregulatory interactions between a Lymphoid and a non-Lymphoid cell*	BioSystems: REACTOME	4.74E−04	1.83E−02	6	136
1270272	Activation of NOXA and translocation to mitochondria	BioSystems: REACTOME	5.21E−04	1.88E−02	2	5
1269102	Nef-mediates down modulation of cell surface receptors by recruiting them to clathrin adapters	BioSystems: REACTOME	5.26E−04	1.88E−02	3	22
M6327	Activation of Csk by cAMP-dependent Protein Kinase Inhibits Signaling through the T Cell Receptor*	MSigDB C2 BIOCARTA (v5.1)	6.84E−04	2.35E−02	3	24
1427859	Cargo recognition for clathrin-mediated endocytosis	BioSystems: REACTOME	7.78E−04	2.58E−02	5	99
137922	*IL12*-mediated signaling events*	BioSystems: Pathway Interaction Database	1.01E−03	3.24E−02	4	61
1269603	Binding of TCF/LEF:CTNNB1 to target gene promoters	BioSystems: REACTOME	1.08E−03	3.24E−02	2	7
137936	*IL12* signaling mediated by STAT4*	BioSystems: Pathway Interaction Database	1.08E−03	3.24E−02	3	28
1269100	The role of Nef in HIV-1 replication and disease pathogenesis*	BioSystems: REACTOME	1.20E−03	3.49E−02	3	29
83004	Propanoate metabolism	BioSystems: KEGG	1.61E−03	4.52E−02	3	32
1269298	Fc epsilon receptor (FCERI) signaling*	BioSystems: REACTOME	1.84E−03	4.94E−02	9	381
117293	Arrhythmogenic right ventricular cardiomyopathy (ARVC)	BioSystems: KEGG	1.88E−03	4.94E−02	4	72
1269528	SMAD2/SMAD3:SMAD4 heterotrimer regulates transcription	BioSystems: REACTOME	1.92E−03	4.94E−02	3	34

Pathways annotated with a * are immune related.

*FDR B&H, false discovery rate using Benjamini–Hochberg method; Genes from input, number of significant genes included in given pathways; Genes in annotation, number of genes involved in functional pathway; MSigDB C2 BIOCARTA, Molecular Signatures Database curated gene set derived from BIOCARTA database; KEGG, Kyoto Encyclopedia of Genes and Genomes*.

**Table 3:** Secreted proteins from meta-analysis and T2 *ad hoc* analysis.

Genes	FC_GSE46510	FC_GSE59491	FC_GSE73685	Directionality	*p*-Value	Adj *p* value
*HPSE*	1.12502566	1.029755132	1.302481986	Upregulated	0.01472767	0.072407187
*NLRP3*	1.120388112	1.045936747	1.463420748	Upregulated	0.008637567	0.055169509
*LRG1*	1.304600844	1.012782905	1.266657092	Upregulated	0.001694397	0.02445227
*CLC*	0.77816604	0.925657768	0.460056969	Downregulated	0.013608709	0.06964973
*DPP4*	0.906503094	0.939578805	0.7254311	Downregulated	0.007598762	0.051200829
IL1R1*	1.088237667	1.085324456	1.324171306	Upregulated	0.006616252	0.047794152
*IL1RAP*	1.163741033	1.016218763	1.310389926	Upregulated	0.017000877	0.07821447
*LAMB2*	1.05178463	1.022848768	1.305601216	Upregulated	0.004096624	0.037683905
*NELL2*	0.785324082	0.91373226	0.642718858	Downregulated	0.000198362	0.010174565
*NUCB2*	0.749613912	0.965573738	0.800992598	Downregulated	0.000657135	0.01646351
*SERPINI1*	0.920203336	0.872420873	0.755891687	Downregulated	0.000180817	0.009596761
TFPI*	1.044097212	1.117168095	0.750437685	Upregulated	0.009325712	0.057394342
*IL1R2*	1.231921476	1.051452709	1.625438109	Upregulated	0.004772589	0.040619724
*CST7*	1.188969828	1.033458464	1.334064851	Upregulated	0.003500353	0.034868265
*CD8B*	0.856761369	0.909815767	0.742642806	Downregulated	0.006032779	0.045913205

Genes with * annotation are also found to be significant in the T2 analysis.

*FC_GSE46510, fold-change calculated using GSE46510 samples; FC_GSE59491, fold-change calculated using GSE59491 samples; FC_GSE73685, fold-change calculated using GSE73685 samples; Adj p val, adjusted p-value*.

These immune pathways and secreted proteins associated with sPTB could have been missed in the single-study analysis due to limited sample size and thus not reaching statistical significance: only 26 significant genes were identified (FDR < 0.10) in the GSE59491 study and no significant genes were identified (FDR < 0.10) in the GSE73685 study (analysis of maternal blood sample only). This highlights the importance and power of aggregating the data and performing a meta-analysis.

### Cell-Type Deconvolution Analysis in Maternal Blood

Due to the heterogeneity of plasma samples, it is important to identify and quantify the various cell types that comprise peripheral blood. If not taken into account, the variability in cell composition in each sample can confound the results and limit interpretability (36). To examine the reproducibility of our experiments and test the hypothesis of aligning our pathways with cell type abundance when comparing preterm and term birth, we performed a cell-type deconvolution analysis. Specifically, since immune cells constitute a large portion of cell types in plasma, and a large part of differentially expressed pathways were immune related, we utilized xCell, a computational method that is able to infer 64 various immune and stroma cell types using gene signatures (37).

**Figure 5:**
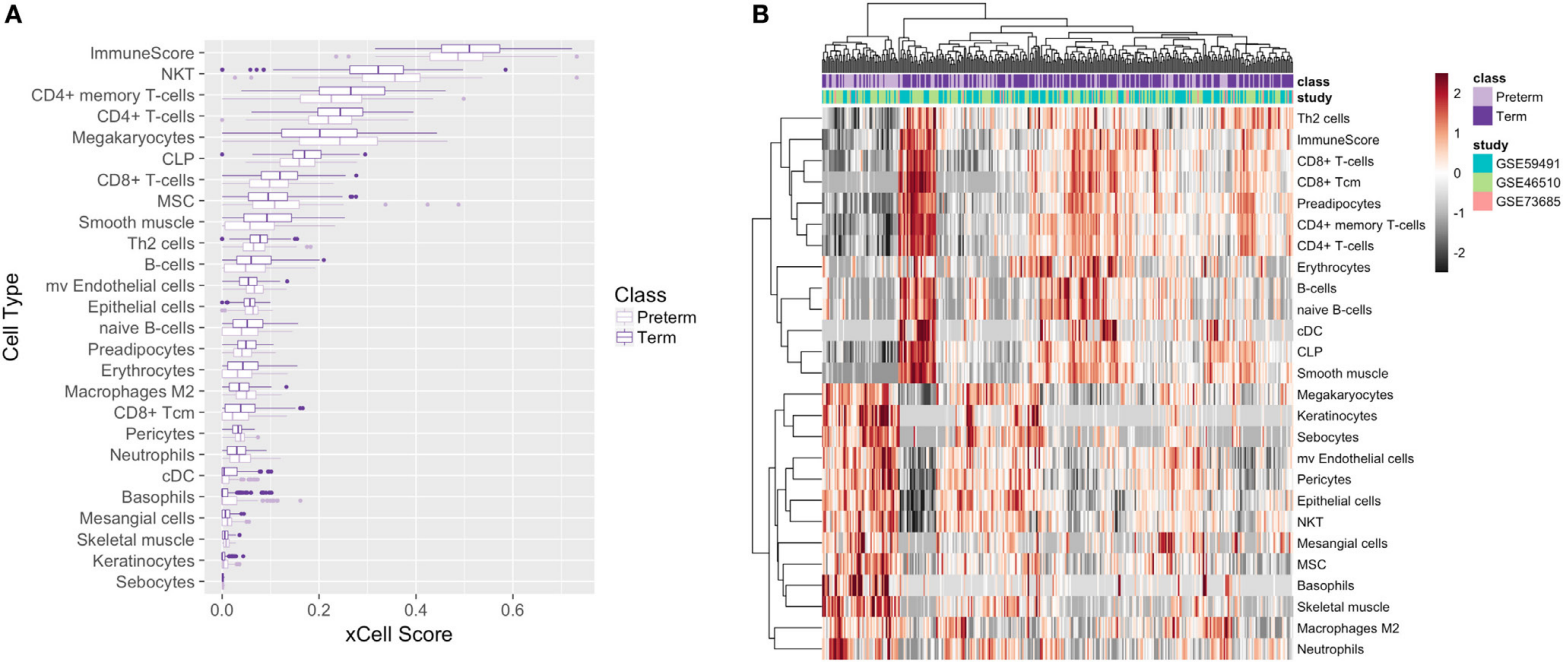
Cell deconvolution of 339 meta-analysis samples. Boxplot **(A)** and heatmap **(B)** of average xCell scores for enriched cell types.

Our xCell analysis of all 339 samples revealed that there were 27 cell types that were enriched and significantly differentially expressed between preterm and term birth. Macrophages (M2 type) and microvascular endothelial cells demonstrated the largest and most significant (FDR = 0.003) difference in enrichment between preterm and term maternal blood samples (Figure 5A) when comparing average xCell scores. We also saw some clustering of preterm and term birth samples by immune cell type, with term birth samples showing some clustering and upregulation of adaptive immune cells, such as Th2 cells, CD8+ T-cells, CD4+ T-cells, and B-cells, and PTB samples showing some clustering and upregulation of innate immune cells, such as NKT, macrophages M2, basophils, and neutrophils (Figure 5B). Adjusting for significant cell types as a covariate in our differential expression analysis for T3 samples resulted in 334 genes that were differentially expressed in PTB compared with term birth. Upon pathway analysis, we found that the innate immune pathway was upregulated in the preterm samples, which is consistent with our initial results.

### Additional Analysis of Maternal Signatures in the Second Trimester

**Figure 6:**
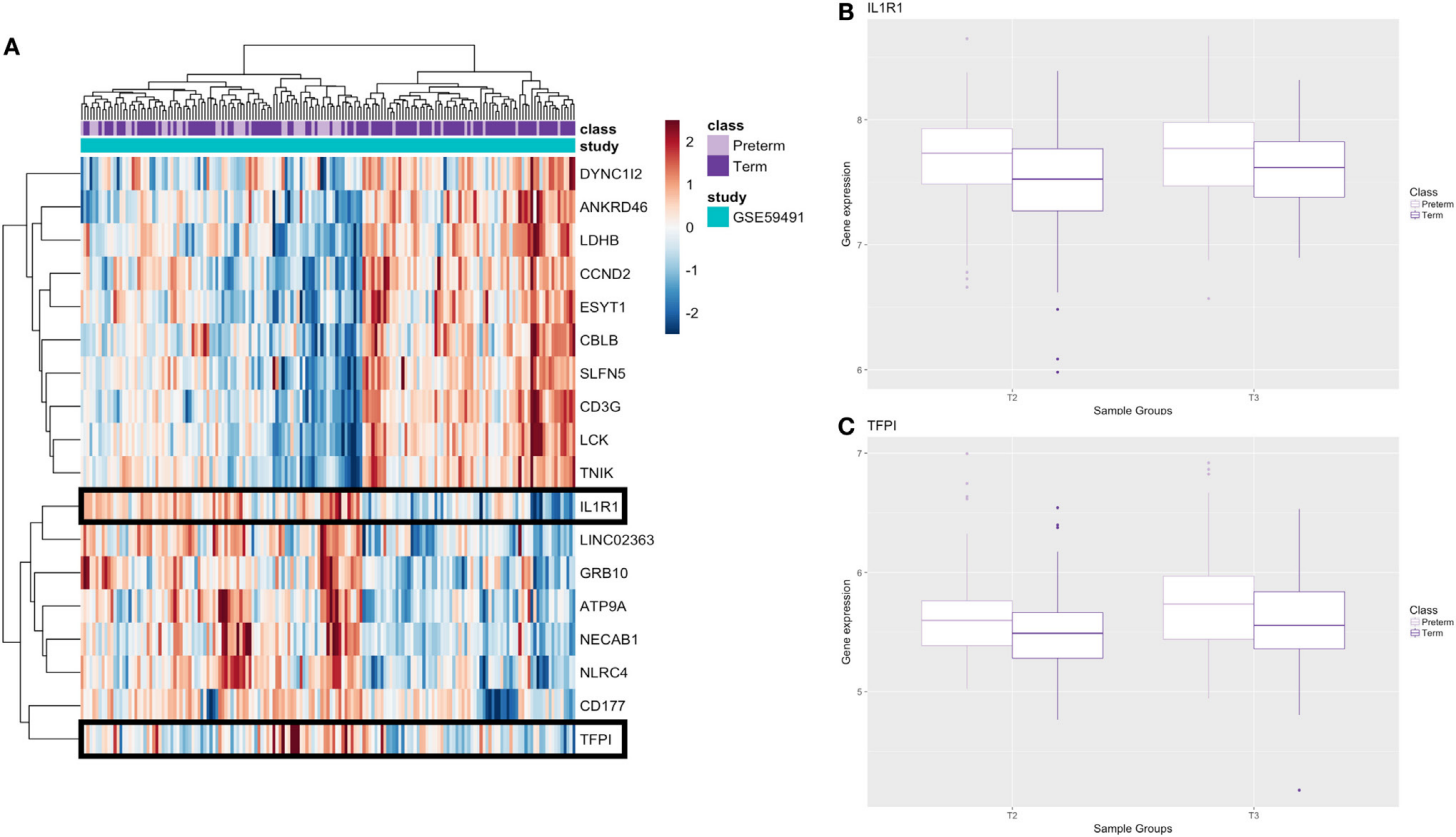
Results from additional second trimester analysis. **(A)** Heatmap of significant genes from second trimester analysis; genes which are secreted as proteins are boxed. **(B,C)** Boxplots of genes that encode secreted proteins at second (T2) and third (T3) trimester; raw gene expression values from GSE59491 are plotted.

To investigate and compare expression profiles at two different time points in pregnancy, we utilized the samples collected at second trimester in the GSE59491 study and performed an additional analysis investigating whether any of our significant genes from the third trimester analysis were differentially expressed at an earlier time point to facilitate potential biomarker identification. Implementing an FDR < 0.1 on the filtered list of 210 genes, there were 18 genes (8 upregulated and 10 downregulated) that were significantly differentially expressed (Figure 6A; Table S3 in Supplementary Material).

These 18 genes, which were differentially expressed in PTB relative to term birth at the second trimester (17–20 weeks) and the third trimester (24–36 weeks), showed similar FC direction and values when comparing second trimester samples from GSE59491 and the samples from the cross-study meta-analysis (Table S4 in Supplementary Material). Furthermore, when plotting the raw expression data for the second and third trimester samples from GSE59491 for these 18 genes, the same trends were upheld, demonstrating similar FC direction and values between the two groups (Figure S1 in Supplementary Material). 2 of these 18 genes (*IL-1R1* and *TFPI*) showed potential as diagnostic biomarkers; they were found to be secreted and detectable in human plasma in the UniProt database (35) (Table 3) and upheld the same fold-change directionality in both second and third trimester samples (Figures 6B,C).

### Upstream Transcription and Cytokine Regulation Analysis in Maternal Signatures

**Figure 7:**
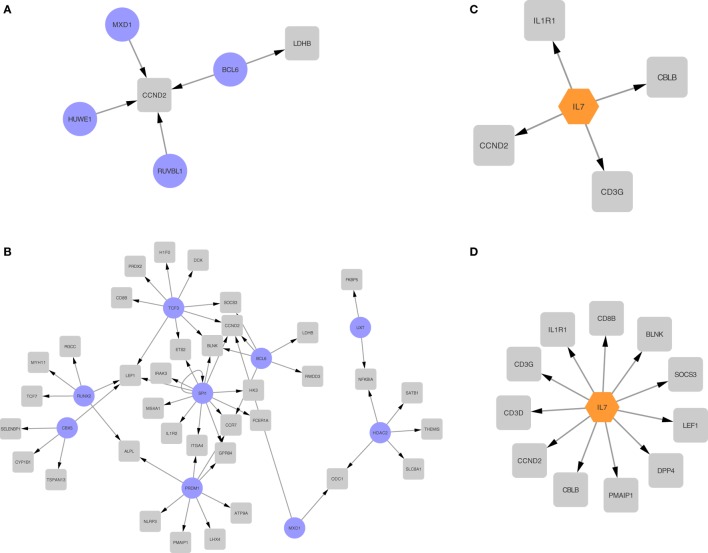
Regulatory networks for second and third trimester differentially expressed genes. Transcription regulation networks for differentially expressed genes in the second trimester **(A)** and third trimester **(B)**, where the transcription factors are represented with a purple round node and the differentially expressed targets are represented with a gray square node. Cytokine networks for second trimester **(C)** and third trimester **(D)**, where the transcription factors are represented with an orange hexagon node and the differentially expressed targets are represented with a gray square node.

To better understand the differential expression patterns, we explored the upstream regulation of differentially expressed upregulated genes for the second and third trimester separately. We first created a transcription factor regulation network for the second and third trimester (Figures 7A,B). In Figure 7A, we found four regulators for only two of the second trimester differently expressed genes. Out of the four regulators, one transcription factor, *BCL6* (38), has been shown before to regulate components of the immune system and another, *MXD1*, is involved in cell proliferation (39). In Figure 7B, we found nine regulators for 46 of the third trimester differently expressed genes. Out of those nine, a few are known to be involved in development of the immune system, such as *SPI1* (40), *BCL6*, and *UXT* (41) while others are involved in embryonic cell development such as *CBX5* (42), *RUNX2* (43), and *TCF3* (44). The overlap between the groups is two transcription factors, *BCL6* and *MXD1*.

We then explored cytokine regulation in differentially expressed upregulated genes in second and third trimesters. In both trimesters, as shown clearly in Figures 7C,D, *IL-7* is the only cytokine we found to be involved in the regulation. Given the known role for *IL-7* signaling in lymphocyte differentiation, this finding is also consistent with the immune signature we observed.

Based on those four regulatory networks and two modes of regulation, we see enrichment of transcription factors involved with the immune system and with cell proliferation.

### Differential Gene Expression Analysis in Samples From Other Tissues

**Figure 8:**
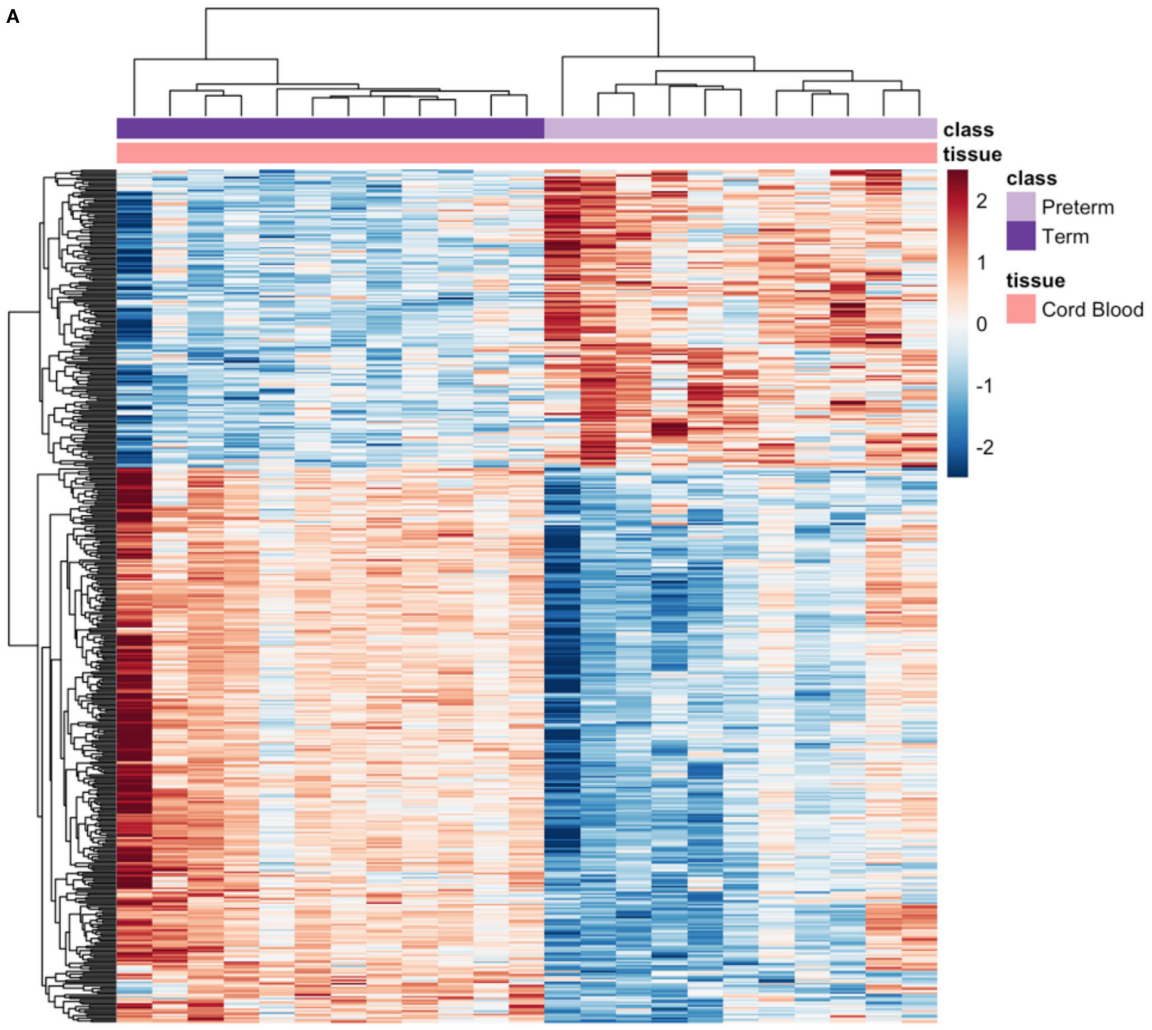

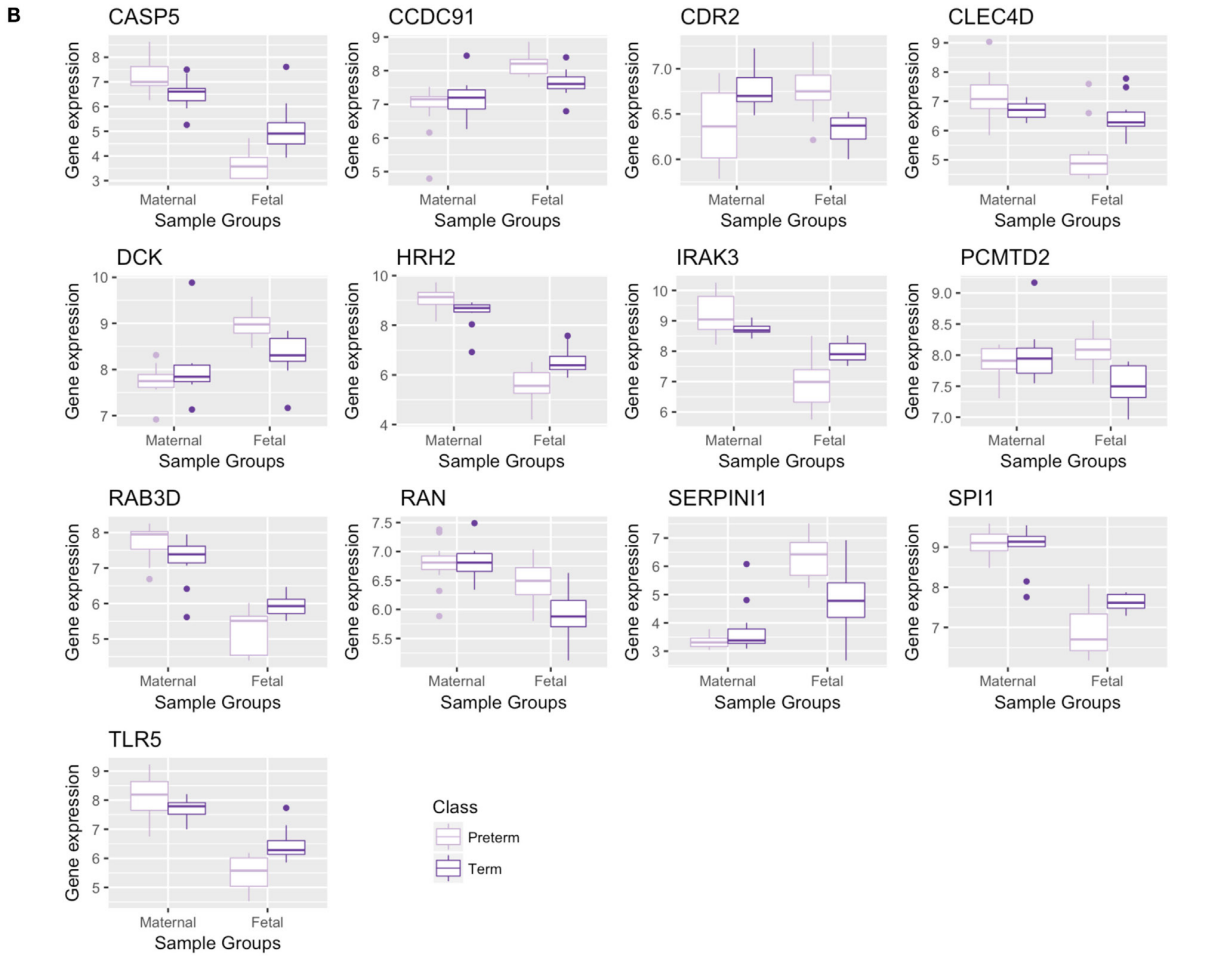
Significant genes from cord blood (CB) tissue analysis and maternal–cord gene signature comparison. **(A)** Heatmap of significant differentially expressed genes from CB analysis. **(B)** Boxplot of overlapping significant genes from meta-analysis and CB analysis; raw gene expression values from GSE73685 plotted.

Since GSE73685 contained a set of diverse tissues, we also evaluated transcriptional signal in various maternal and fetal tissues separately. With an FDR < 0.05, only one of the tissue types, CB, showed significant differentially expressed genes. Imposing a fold-change cutoff of 1.3 on the 507 genes that were identified from the differential expression analysis resulted in 473 significant genes (Table S5 in Supplementary Material), 165 upregulated and 308 downregulated genes in PTB relative to term birth, which clustered to create a distinct separation between PTB and term birth (Figure 8A). Based on the ToppFun database, 308 downregulated genes were highly enriched in multiple functional pathways, many of which were immune related (Table 4) (34). Specifically, PTB samples showed downregulation of many innate immune-related pathways relative to term birth samples. Conversely, the 165 upregulated genes showed low-functional pathway enrichment (Table 4) (34).

**Table 4:** Functionally enriched pathways from cord blood tissue analysis.

ID	Name	Source	*p*-Value	FDR B&H	Genes from input	Genes in annotation
**Upregulated**						
169351	Validated targets of *C-MYC* transcriptional activation	BioSystems: Pathway Interaction Database	7.733E−08	0.00008437	9	81
**Downregulated**						
1269203	Innate Immune System*	BioSystems: REACTOME	7.768E−26	9.578E−23	81	1312
1457780	Neutrophil degranulation*	BioSystems: REACTOME	8.786E−25	5.417E−22	50	492
213780	Tuberculosis*	BioSystems: KEGG	5.678E−07	0.0002334	15	179
83051	Cytokine-cytokine receptor interaction*	BioSystems: KEGG	0.000001234	0.0003804	18	270
469200	Legionellosis*	BioSystems: KEGG	0.000004663	0.0009906	8	55
144181	Leishmaniasis*	BioSystems: KEGG	0.00000482	0.0009906	9	73
1427857	Regulation of *TLR* by endogenous ligand*	BioSystems: REACTOME	0.000005868	0.001034	5	16
M9546	Chaperones modulate interferon Signaling Pathway*	MSigDB C2 BIOCARTA (v5.1)	0.00001497	0.002122	5	19
1269204	Toll-Like Receptors Cascades*	BioSystems: REACTOME	0.00001549	0.002122	12	153
1269310	Cytokine Signaling in Immune system*	BioSystems: REACTOME	0.00002554	0.002599	30	763
1269158	*IRAK4* deficiency (*TLR2/4*)*	BioSystems: REACTOME	0.0000274	0.002599	4	11
PW:0000234	Innate immune response*	Pathway Ontology	0.0000274	0.002599	4	11
1269160	*MyD88* deficiency (*TLR2/4*)*	BioSystems: REACTOME	0.0000274	0.002599	4	11
634527	*NF-kappa B* signaling pathway*	BioSystems: KEGG	0.00004183	0.003636	9	95
122191	NOD-like receptor signaling pathway*	BioSystems: KEGG	0.00004423	0.003636	12	170
1269156	Diseases of Immune System*	BioSystems: REACTOME	0.00005093	0.003694	5	24
1269157	Diseases associated with the *TLR* signaling cascade*	BioSystems: REACTOME	0.00005093	0.003694	5	24
1269318	Signaling by Interleukins*	BioSystems: REACTOME	0.00005712	0.003913	23	531
M13968	HIV-I Nef: negative effector of Fas and TNF*	MSigDB C2 BIOCARTA (v5.1)	0.00006456	0.00419	7	58
138052	Ephrin B reverse signaling	BioSystems: Pathway Interaction Database	0.00009269	0.005531	5	27
193147	Osteoclast differentiation	BioSystems: KEGG	0.0000942	0.005531	10	130
1383066	*TP53* Regulates Transcription of Cell Death Genes	BioSystems: REACTOME	0.0001239	0.006944	6	45
P00031	Inflammation mediated by chemokine and cytokine signaling pathway*	PantherDB	0.0001357	0.007273	12	191
1269545	Class A/1 (Rhodopsin-like receptors)	BioSystems: REACTOME	0.0001744	0.00896	16	322
1269236	Activated *TLR4* signaling*	BioSystems: REACTOME	0.0001852	0.009001	9	115
114228	Fc gamma R-mediated phagocytosis*	BioSystems: KEGG	0.0001898	0.009001	8	91
217173	Influenza A*	BioSystems: KEGG	0.0002322	0.01017	11	173
1269239	Toll-Like Receptor *TLR1*:*TLR2* Cascade*	BioSystems: REACTOME	0.0002557	0.01017	8	95
1269238	Toll-Like Receptor 2 (*TLR2*) Cascade*	BioSystems: REACTOME	0.0002557	0.01017	8	95
1269237	*MyD88*:Mal cascade initiated on plasma membrane*	BioSystems: REACTOME	0.0002557	0.01017	8	95
1269240	Toll-Like Receptor *TLR6*:*TLR2* Cascade*	BioSystems: REACTOME	0.0002557	0.01017	8	95
137995	HIV-1 Nef: Negative effector of Fas and TNF-alpha*	BioSystems: Pathway Interaction Database	0.0003326	0.01282	5	35
99051	Chemokine signaling pathway*	BioSystems: KEGG	0.0003596	0.01294	11	182
137910	*CXCR4*-mediated signaling events*	BioSystems: Pathway Interaction Database	0.0003598	0.01294	7	76
1269234	Toll-Like Receptor 4 (*TLR4*) Cascade*	BioSystems: REACTOME	0.0003674	0.01294	9	126
147809	Chagas disease (American trypanosomiasis)*	BioSystems: KEGG	0.0004156	0.01403	8	102
172846	Staphylococcus aureus infection*	BioSystems: KEGG	0.0004209	0.01403	6	56
1457777	Antimicrobial peptides*	BioSystems: REACTOME	0.0005053	0.0164	8	105
1269280	*FCGR* activation*	BioSystems: REACTOME	0.0005221	0.01651	4	22
213306	Measles*	BioSystems: KEGG	0.0005771	0.01779	9	134
M15285	*NF-kB* Signaling Pathway*	MSigDB C2 BIOCARTA (v5.1)	0.0006234	0.01875	4	23
138022	Class I *PI3K* signaling events	BioSystems: Pathway Interaction Database	0.0007051	0.02047	5	41
83060	Apoptosis	BioSystems: KEGG	0.0007139	0.02047	9	138
375172	*Salmonella* infection*	BioSystems: KEGG	0.0007631	0.02138	7	86
169642	Toxoplasmosis*	BioSystems: KEGG	0.0008232	0.02256	8	113
1269161	*MyD88* deficiency (*TLR5*)*	BioSystems: REACTOME	0.0009052	0.02375	2	3
1269566	Hydroxycarboxylic acid-binding receptors	BioSystems: REACTOME	0.0009052	0.02375	2	3
1269303	C-type lectin receptors (CLRs)*	BioSystems: REACTOME	0.00112	0.02877	9	147
137964	Regulation of p38-alpha and p38-beta*	BioSystems: Pathway Interaction Database	0.00117	0.02937	4	27
1269576	G alpha (i) signaling events	BioSystems: REACTOME	0.001191	0.02937	12	243
1270241	Signal regulatory protein (*SIRP*) family interactions*	BioSystems: REACTOME	0.00133	0.03216	3	13
1269308	Dectin-2 family*	BioSystems: REACTOME	0.00154	0.03607	4	29
153910	Phagosome*	BioSystems: KEGG	0.001551	0.03607	9	154
1470924	Interleukin-10 signaling*	BioSystems: REACTOME	0.001602	0.03617	5	49
1269546	Peptide ligand-binding receptors	BioSystems: REACTOME	0.001752	0.03617	10	188
1269332	*TNF*s bind their physiological receptors*	BioSystems: REACTOME	0.001753	0.03617	4	30
137974	Caspase cascade in apoptosis*	BioSystems: Pathway Interaction Database	0.001755	0.03617	5	50
138017	Signaling events mediated by *PTP1B*	BioSystems: Pathway Interaction Database	0.001755	0.03617	5	50
PW:0000681	FasL mediated signaling pathway*	Pathway Ontology	0.001789	0.03617	2	4
1269159	*IRAK4* deficiency (*TLR5*)*	BioSystems: REACTOME	0.001789	0.03617	2	4
PW:0000464	leukotriene metabolic*	Pathway Ontology	0.001789	0.03617	2	4
83099	Amyotrophic lateral sclerosis (ALS)	BioSystems: KEGG	0.001919	0.03816	5	51
P00020	*FAS* signaling pathway	PantherDB	0.001986	0.03874	4	31
M17681	*IL3* signaling pathway*	MSigDB C2 BIOCARTA (v5.1)	0.002062	0.03874	3	15
M11736	Cytokines can induce activation of matrix metalloproteinases, which degrade extracellular matrix*	MSigDB C2 BIOCARTA (v5.1)	0.002062	0.03874	3	15
P00006	Apoptosis signaling pathway	PantherDB	0.002073	0.03874	7	102
1269195	Antigen processing-Cross presentation*	BioSystems: REACTOME	0.002192	0.04034	7	103
137939	Direct *P53* effectors	BioSystems: Pathway Interaction Database	0.002234	0.04051	8	132
1269357	GPVI-mediated activation cascade*	BioSystems: REACTOME	0.002476	0.04291	5	54
M14775	G alpha s Pathway	MSigDB C2 BIOCARTA (v5.1)	0.002506	0.04291	3	16
1270299	*RIPK1*-mediated regulated necrosis	BioSystems: REACTOME	0.002506	0.04291	3	16
1270298	Regulated Necrosis	BioSystems: REACTOME	0.002506	0.04291	3	16
1269544	*GPCR* ligand binding	BioSystems: REACTOME	0.0027	0.04561	17	455
812256	*TNF* signaling pathway*	BioSystems: KEGG	0.002867	0.04778	7	108
M4891	Regulation of transcriptional activity by PML*	MSigDB C2 BIOCARTA (v5.1)	0.003003	0.04873	3	17
1270264	Ligand-dependent caspase activation*	BioSystems: REACTOME	0.003003	0.04873	3	17
194384	African trypanosomiasis*	BioSystems: KEGG	0.003129	0.04946	4	35
137944	*IL1*-mediated signaling events*	BioSystems: Pathway Interaction Database	0.003129	0.04946	4	35

Pathways annotated with a * are immune related.

*FDR B&H, FDR using Benjamini–Hochberg method; Genes from input, number of significant genes included in given pathways; Genes in annotation, number of genes involved in functional pathway, MSigDB C2 BIOCARTA, Molecular Signatures Database curated gene set derived from BIOCARTA database; KEGG, Kyoto Encyclopedia of Genes and Genomes; PANTHER, Protein Analysis Through Evolutionary Relationships Classification system*.

Comparing these 473 significant genes from the CB analysis to the 210 significant genes output from the maternal blood meta-analysis, we found that there were 13 genes including toll-like receptor 5 (*TLR5*) and other immune transcripts which overlapped and were significant in both analyses. Plotting the raw data for these 13 genes from GSE73685 revealed opposite directionality comparing preterm and term birth for CB and maternal blood, respectively (Figure 8B). While some genes were upregulated in preterm maternal whole blood samples (in both the meta-analysis and GSE73685 only samples), those same genes were downregulated in preterm CB samples; the same was true for many genes which were downregulated in preterm maternal whole blood samples but upregulated in preterm CB samples.

All the results and the data are available as an RShiny Application for the benefit of the research community: http://comphealth.ucsf.edu/preterm_transcriptomics/.

## Discussion

Given the role of the immune system in pregnancy, there exists a need to elucidate immune signatures specific to PTB at both the maternal and fetal level. This study was thus designed to answer these questions by aggregating data from multiple independent experiments in an attempt to discover significant, differential genetic signatures in women who deliver preterm. Our cross-study meta-analyses revealed 210 differentially expressed genes, 15 of which were found to be secreted in the plasma. Interestingly, 18 of these 210 genes also demonstrated differential expression in the second trimester, suggesting a possibility for early identification of patients who might deliver preterm. *IL-1R1* and *TFPI*, both of which encode immune-related proteins, were found to be differentially expressed and secreted longitudinally. CB analysis also revealed significant differential gene expression and had clustering in immune related pathways. In contrast to preterm maternal whole blood, which showed upregulation of innate immunity and downregulation of adaptive immunity, CB showed downregulation in innate immunity. This juxtaposition, as well as the heavy involvement of immune-related pathways and biomarkers, bring to light novel findings which coincide with previous literature.

### Leveraging Transcriptomics to Identify New Biomarkers for sPTB

There is a crucial need to find biomarkers for PTB. There are classic negative predictors such as the absence of fetal fibronectin in the cervicovaginal fluid, but they are less useful as a routine screening tool to identify women with high risk of PTB (45–47). Identifying biomarkers predictive of PTB in maternal blood seems like an easier target as blood is easily accessible and can be collected in most women as part of the standard prenatal care (27). In our study, we found nine upregulated genes that encode secreted proteins in human (48). These markers may be further investigated regarding their values as biomarkers for identifying high-risk women for PTB, especially *IL-1R1* and *TFPI* that were significantly over expressed among PTB cases as early as during second trimester.

IL-1 receptor type I belongs to the *IL-1* family of receptors which contains 10 distinct but related gene products all of which are heavily involved in the innate immune response. This receptor has a variety of ligands which are involved in the initiation (*IL-1α* and *IL-1β*) and inhibition (*IL-1Ra*) of the immune and inflammatory responses (49). *IL-1α* belongs to a group of dual-function cytokines, constitutively present inside cells under normal homeostatic condition and playing a role as a transcription regulator to trigger inflammation and immunity extracellularly (50). This ligand has been shown to induce an inflammatory response in absence of infection as well as is responsible for the stimulation and release of *IL-1β* from monocytes (51). Conversely, *IL-1β* is not expressed in homeostatic conditions and is active only upon cleavage of its precursor caspase-1 (50). Although *IL-1Iα* is the initiator of sterile inflammation *IL-1β* has been shown to play a role as an amplifier of inflammation (50, 51). The binding of either of these molecules to *IL-1R1* leads to the activation of many transcription factors including nuclear factor-kappa B (*NF-kB*) and ultimately leads to an inflammatory response (49).

IL-1 receptor type I has been studied as one of the potential biomarkers to predict heart failure in hypertensive patients (52) and was proposed as a candidate molecular target for rheumatoid arthritis treatment (53). In the pregnancy space, *IL-1R1* has been investigated in endometrial tissues and chorioamnionitis (54, 55) and has been found to be increased in PTBs stimulated by RU486 in rats (56). One study found an aberrant placental expression of interleukin 1 receptor-like 1 (*IL-1RL1*) in PTB cases (compared with spontaneous term births) whose mRNA transcript were of higher detection in maternal plasma samples than their gestational age-matched controls that had term birth, suggesting *IL-1RL1* to be a candidate PTB-associated marker (57). Other cytokines have also been identified as PTB biomarkers, including *IL6, IL-1β*, and *IL2* (26). In case of infection, blocking a single factor on the pathway may not be sufficient to prevent preterm delivery (58). Our finding suggests that *IL-1R1* could be one of the detectable markers of the dysregulated inflammatory network associated with PTB that bears further investigation.

The overall signature we observed is consistent with previously published literature supporting a role for the inflammasome and activation of the innate immune system in the onset of spontaneous preterm labor. For example, activation of the *NLRP3* inflammasome, which ultimately results in increased levels of mature *IL-1β*, has also been implicated in patients (59). There are increased levels of *IL-1β* in the amniotic fluid of patients with preterm labor (60) as well as in the chorioamniotic membranes (59). A GWAS study also reported that polymorphisms in the *IL1R* antagonist locus were associated with PTB (61, 62). In mouse models, introduction of *IL1* can induce PTB by activating the innate immune system, and blockade of *IL1R* can abrogate this phenotype (63). Given the extensive downstream effects of this signaling pathway in influencing neonatal morbidity in preterm infants (64), our findings have clinical relevance for discovering targetable molecular pathways.

Tissue factor is a key element for normal gestation (65). Maternal plasma concentrations of total *TFPI*, the main physiological inhibitor of the tissue factor-dependent pathway of blood coagulation, is shown to increase during the first half of pregnancy, remain relatively constant in the remaining half, and decrease during labor (66–68). Different profiles of maternal plasma tissue factor and *TFPI* concentrations have been observed among several obstetrical syndromes including preeclampsia (69), preterm prelabor rupture of membranes (70), and small for gestational age (69).

### Maternal and Fetal Signals Elucidate the Role of Adaptive and Innate Immunity in PTB

Immunity and inflammation have been shown to play an important role in parturition timing (71–75). Specifically, infection and breakdown of maternal–fetal tolerance (rejection) are the two most important in this respect. These have different association with gestational age, with infection (76) affecting mainly early PTB while rejection (77) affecting mainly late PTB cases. Healthy pregnancy involves multiple tolerance mechanisms that prevent the maternal and fetal immune systems from recognizing and rejecting each other (78, 79), whereas preterm labor may result from a breakdown in maternal–fetal tolerance (12). Kourtis et al. conclude that aspects of innate immunity are maintained or enhanced during pregnancy, particularly during the second and third trimesters and there are decreases in adaptive immunity seen in later stages of pregnancy (80). Before labor, the maternal immune system modulates inflammatory signaling pathways to avoid rejection of the fetus. Conversely, in pregnancies with PTB, the fetal immune system might undergo activation, resulting in recognition and rejection of maternal antigens. Implications of pregnancy as a modulated immunological condition are vast including prevention of fetal rejection, susceptibility to some infections and maybe even PTB (80).

The upregulated and downregulated gene signatures identified in the maternal meta-analysis demonstrate a clear enrichment in immune-related pathways. When looking at the regulation of the differentially expressed genes, we found that transcription factors regulating the differentially expressed genes were also immune-related transcription factors. Looking at cytokine data, we found *IL-1*-related pathways are upregulated during the third trimester for women who deliver preterm. This supports the upregulation of inflammatory pathways involving cytokines and their receptors among PTB cases reported by Heng et al.’s (28) study, whose data were included in the current meta-analysis. Genes encoding *IL-1α* and *IL-1β*, two founding members of the *IL-1* family that have played a central role in several autoinflammatory diseases (81–83), and other cytokines such as *IL-6* are also upregulated in our study, despite not being statistically significant after multiple testing correction. Past research suggests that pro-inflammatory cytokines *IL-1β* and *TNF-α* play a primary role in inducing infection-associated PTB (58, 84). These findings are consistent with the literature and are more reflective of early rather than late PTB. The upregulated inflammatory-related pathways in this study may be in part attributed to clinical or sub-clinical infection. However, diagnosis of infection is often not available in population studies, which precludes further exploration of the contribution of infection in the observed signal.

Based on the maternal data, we found that genes and cell types associated with innate immunity were upregulated in PTB while those relevant to adaptive immunity were downregulated in PTB. Genes identified in the fetal CB analysis showed enrichment in pathways that were immune related but the signature was flipped; innate immunity was downregulated in babies born preterm. One hypothesis is that the immune systems of women who deliver preterm are less responsive to specific foreign antigens such as infections which themselves could lead to PTB, while mothers whose adaptive immunity was stronger were able to maintain the pregnancy due to better immune coping mechanisms. Previously, polymorphisms of genes pertaining to the innate immune system were found to have only moderate effects on subsequent PTB, although they played a functionally relevant role in host immune response (85). On the other hand, babies that were born preterm showed a downregulation of innate immunity, which suggests opposing signals in the maternal and fetal immune tolerance but also could be a result of the incomplete development of immune defense. Since innate immunity serves as the first defense of the human immune systems, weaker innate immunity signals could be indicative of vulnerability and susceptibility to life-threatening infections (86). There is some evidence that this homeostasis of the fetal–maternal immune tolerance can be perturbed during infection, resulting in immune activation and the observed opposing signals can be indicative of the breakdown of the tolerance mechanism leading up to PTB.

Specifically, there are several genes that are reversed in the maternal and fetal signatures. *TLR5* was one of the genes we found to have opposing differential gene expression when comparing mother and fetus. While *TLR5* showed lower expression in PTB CB samples, *TLR5* was upregulated in PTB maternal whole blood samples. *TLR5*, as well as other toll-like receptors, play an important role in pathogen recognition and subsequent activation of the inflammatory innate immune response. *TLR5* (along with *TLR2* and *TLR3*) has previously been implicated in regulation of pro-inflammatory and pro-labor responses in primary human myometrium cells (87). One of the downstream targets of this gene in the *MyD88*-dependent pathway is *NF-kB*, a critical transcription factor in the activation of genes related to immune and inflammatory responses (88–90). *TLR5* has also been shown to increase production of various pro-inflammatory interleukins including *IL-6* and *IL-8* (87, 91).

Importance of *TLR5* in pregnancy and its association with PTB has been shown repeatedly. The *TLR5* (g.1174C>T) variant, which encodes a non-functional protein, is significantly associated with development of severe bronchopulmonary dysplasia in very low-birth weight infants born prematurely. This evidence shows that the non-functionality of *TLR5* in preterm infants results in an insufficient immune response to flagellated bacteria (92). Furthermore, *TLR5* mRNA expression has repeatedly been found to be increased in the placenta following spontaneous term labor (91, 93).

This study has several limitations that may be encountered in other similar studies. First, we were limited to the number of studies with publicly available data that could be aggregated together for our meta-analysis. In addition, a common shortcoming of using publicly available data is that samples lacked demographic information as well as detailed clinical annotations. Furthermore, samples included in our study are heterogeneous as they came from studies with different design (cohort or case–control), phenotype—late and early PTB, and different populations (dataset GSE46510 consisting samples from women with threatened preterm labor). Yet, the current comparison between sPTB cases and term birth controls were likely to be an underestimation of the underlying different gene expression profile between the two groups due to the inclusion of symptomatic women non-differentially as both cases and controls (increasing the baseline risk of sPTB among the controls) as well as confounding factors such as infection and other obstetric complications. In addition, although we propose several potential novel biomarkers, our data are limited in discerning whether the differential expression signatures observed reflect the membrane bound proteins or their secreted isoforms. However, despite these drawbacks, this paper presents novelty in being the largest published meta-analysis of PTB transcriptomics using publicly available data to date. Since PTB samples are difficult to obtain, the ability to aggregate data by using standardized methods to correct for heterogeneity is exciting since it increases our statistical power and, as a result, allows for the discovery of novel pathways and biomarkers. For example, the pathways associated with sPTB and potential biomarkers for indication of early switch to a pro-inflammatory state of the maternal immune system could have been missed in the single-study analysis due to not reaching statistical significance: only 26 significant genes were identified (FDR < 0.10) in the GSE59491 study and no significant genes were identified (FDR < 0.10) in the GSE73685 study (analysis of maternal blood sample only). Although additional validation is needed, we hope that this paper informs the design and interpretation of clinical biomarker studies. Furthermore, we hope that this meta-analysis incentivizes others to add their data to public repositories with the goal of creating a more comprehensive database for PTB.

This paper presents several future directions including validation of the observed cell type signals through methods such as flow cytometry and CyTOF (94) as well as further exploring the presented transcriptomic signatures for diagnostics and therapeutics. We may be able to validate *TFPI* and *IL-1R1* in additional datasets collected prospectively in combination with clinical data and direct analysis of cell types to correlate with findings in plasma. In addition, staining and imagining of these two proteins in preterm and term whole blood samples can elucidate their sub-cellular location and potential as a clinical biomarker. This could lead to additional large animal studies to identify pathways whose inhibition could be beneficial and efficacious, similar to *IL1* signaling blocked by Anakinra in rheumatoid arthritis. Furthermore, although we evaluated the effect of cell type proportions as a covariate for our differential expression analysis, future studies involving single cell or sorted cell analysis will be much more informative.

## Conclusion

Overall, our comprehensive analysis using publicly available data was able to elucidate genetic signatures associated with sPTB as well as identify potential biomarkers that could be translated to clinical practice. The novel finding of the reversal of regulation in innate immunity in maternal blood samples relative to fetal blood samples in PTB brings to light potential mechanisms that may be at play, which may allow for the prediction of sPTB as well as the development of therapeutics to extend pregnancy to term. In addition, the identification of two potential biomarkers, such as *TFPI* and *IL-1R1*, which are differentially expressed starting at mid-gestation, allows the possibility for clinically diagnostic biomarkers which may identify women at risk for PTB.

## Materials and Methods

### Study Design

The purpose of this study was to perform a cross-study meta-analysis using multiple independent datasets to identify differential expressed genes comparing mothers who deliver preterm to mothers who deliver at term using maternal whole blood samples. Additional analyses across different time points and various tissue types were also performed to investigate differential expression between these two groups (Figure 1).

We searched the NCBI GEO database for public human microarray genome-wide expression studies using search terms including PTB and premature (23, 24). Abstracts were screened and only studies that met the following criteria were included: (i) had both spontaneous preterm cases and term delivery controls in the same study, (ii) included samples collected before or at delivery, and (iii) had a sample size of 20 or more. We used samples from maternal blood for our main analysis as they have the most samples. Classification of samples as PTB or term birth was extracted from sample matrices downloaded from the GEO database.

### Cross-Study Meta-Analysis

We implemented the meta-analysis pipeline by Hughey and Butte (21) for data processing and normalization. All microarray data were renormalized from raw data and merged based on genes meeting two criteria: those with non-missing values and those which were mutually inclusive across all three studies. The merged dataset was subsequently corrected for study-specific effects using ComBat, which implements an empirical Bayes method to correct for study-specific biases and batch effects by performing cross-study normalization (29). An *F*-test was performed to test the equality of variance across the three studies. Differential gene expression analysis was performed on this normalized merged dataset of genes to obtain significance level (*p*-values) of each gene using the R package limma which fits linear models to expression data for each gene (95). We corrected for multiple hypothesis testing using the Benjamini and Hochberg’s (i.e., FDR) method (96) with a pre-specified cutoff of 0.1 to identify more significant genes.

Effect size of each gene is expressed in FC, which was calculated for each study separately using the raw expression data before ComBat. Samples were divided with respect to preterm or term delivery and mean gene expression was calculated for each gene meeting the FDR < 0.1 cutoff. The logged (base 2) average expression values were used to calculate fold-change [FC = 2^(average expression for preterm samples − average expression for term samples)^]. We further filtered the significant genes that met the FDR < 0.1 criteria using a significance threshold at a FC > 1.3 for upregulated genes or <1/1.3 for downregulated genes (22). We obtained a list of genes that showed the largest fold-change and were most differentially expressed, when comparing preterm and term births in the respective study. A final gene set was compiled by combining the significant genes from all three studies; if a gene met the FC cutoff of 1.3 in at least one of the studies, it was included in the final gene data set.

To investigate the relevance of these results, we performed a gene list functional enrichment analysis using ToppFun (34) to identify the pathways our genes were involved in and met a cutoff of FDR < 0.05, evaluated connectivity using the STRING database (31–33), explored biomarker identification using the UniProtKB database (35), and executed a cell type enrichment analysis using xCell (37). We extracted the significant cell types from the xCell output by performing a Student’s two-sided *t*-test and subsequent Benjamini and Hochberg’s (96) multiple testing correction (FDR < 0.05). A more stringent cutoff was used for cell deconvolution to extract the cell types that were robustly, significantly different between the two groups.

We utilized samples collected at the second trimester from GSE59491 (28) and samples from multiple tissues from GSE73685 and performed (1) single-study analysis and (2) tissue-level analyses to further investigate the common differentially expressed gene signatures across time points and different tissue types.

### Single-Study Second Trimester Analysis

To perform a single-study gene expression analysis on the second trimester samples from GSE59491, we merged the gene expression values across all studies, extracted the second trimester samples from GSE59491, and implemented a linear model on that subset of samples. After calculating the *p*-value for each gene, we filtered our list of genes using the output from the cross-study meta-analysis done prior; this resulted in a list of overlapping set of genes which were previously found to be significant in the third trimester. To determine whether these genes were significantly differentially expressed in the second trimester as well, we corrected the raw *p*-values for multiple hypothesis testing for this subset of genes using the Benjamini and Hochberg’s method and imposed an FDR < 0.1 (96).

As an exploratory analysis, we input the resulting genes from the FDR < 0.1 cutoff into the UniProtKB database to determine which genes are secreted as proteins in humans (35).

### Regulatory Network Analysis

Transcription factor regulation networks and cytokine networks were analyzed through the use of Upstream Regulator analytic in IPA (QIAGEN Inc., https://www.qiagenbioinformatics.com/products/ingenuitypathway-analysis). Only significant connections are included in our networks and all connections are based on prior knowledge in IPA Knowledge Base. The transcription factors were filtered and only those that were expressed in a specific trimester were kept, to allow as much accuracy in results as possible.

### Tissue-Level Analyses

To evaluate differential gene expression at a higher resolution, we performed gene expression analyses at an individual tissue level. Combining all tissue data into a merged dataset with all eight tissue types based on mutual genes, we extracted each tissue and created eight tissue-specific datasets for linear model fitting using limma (95). After correcting the raw *p*-values from limma using the Benjamini–Hochberg method for multiple hypotheses testing, seven of the eight tissues did not show significantly differentially expressed genes after implementing an FDR < 0.05; however, one tissue type, CB, had an output of genes which met the FDR criteria (96). A more stringent FDR cutoff was used to delineate the genes with the strongest differential expression between the two groups since an FDR < 0.1 resulted in 1,035 differentially expressed genes. These genes were further filtered by imposing a fold-change cutoff of 1.3 (22) which resulted in a list of significantly differentially expressed genes; the relevance of these genes was explored by performing pathway analysis using ToppFun in which a FDR < 0.05 was used as a cutoff (34).

All the results and the data are available as an RShiny Application for the benefit of the research community: http://comphealth.ucsf.edu/preterm_transcriptomics/.

## Data Availability Statement

The datasets analyzed for this study can be found in the National Institute of Health Gene Expression Omnibus (https://www.ncbi.nlm.nih.gov/geo/). All datasets as well as the results generated in this study are available on the RShiny application (http://comphealth.ucsf.edu/preterm_transcriptomics/) and the ImmPort database (accession: SDY1327).

## Author Contributions

BV, MS, and AW conceived of the study. BV, AW, and IK carried out data analysis. IP carried out data visualization and app development. All authors contributed to writing and editing the manuscript.

## Conflict of Interest Statement

The authors declare that the research was conducted in the absence of any commercial or financial relationships that could be construed as a potential conflict of interest.
