# Supplementary material for: Meta-Analysis of Maternal and Fetal Transcriptomic Data Elucidates the Role of Adaptive and Innate Immunity in Preterm Birth
Source: Front Immunol. 2018 May 9;9:993. doi: 10.3389/fimmu.2018.00993 (PMC5954243; doi:10.3389/fimmu.2018.00993)
Supplement: Supplementary file 1 [file Table_1.docx]

| **Genes** | **FC_GSE46510** | **FC_GSE59491** | **FC_GSE73685** | **Directionality** | **P.Value** | **adj.P.Val** |
| --- | --- | --- | --- | --- | --- | --- |
| **ATP9A** | 1.30090274 | 1.085462301 | 1.228970959 | Upregulated | 9.79E-04 | 1.92E-02 |
| **RASGRP1** | 0.795432511 | 0.947938165 | 0.725856817 | Downregulated | 1.03E-03 | 1.97E-02 |
| **CD96** | 0.854534459 | 0.94108623 | 0.756123635 | Downregulated | 7.88E-04 | 1.77E-02 |
| **CDR2** | 0.864691483 | 0.941956056 | 0.75019476 | Downregulated | 6.98E-04 | 1.69E-02 |
| **VTI1B** | 0.863876539 | 0.992982342 | 0.767287933 | Downregulated | 2.37E-02 | 9.35E-02 |
| **ANP32B** | 0.823580958 | 1.007126822 | 0.725354209 | Downregulated | 5.65E-04 | 1.54E-02 |
| **CETN2** | 0.895869666 | 0.975133817 | 0.745513366 | Downregulated | 2.40E-03 | 2.88E-02 |
| **GRAP** | 0.954480836 | 0.951377503 | 0.709571948 | Downregulated | 1.04E-02 | 6.04E-02 |
| **HPSE** | 1.12502566 | 1.029755132 | 1.302481986 | Upregulated | 1.47E-02 | 7.24E-02 |
| **DBF4** | 0.754517567 | 0.950425555 | 0.852149145 | Downregulated | 2.35E-04 | 1.09E-02 |
| **IRAK3** | 1.121103533 | 1.040570984 | 1.412199322 | Upregulated | 2.26E-02 | 9.10E-02 |
| **NLRP3** | 1.120388112 | 1.045936747 | 1.463420748 | Upregulated | 8.64E-03 | 5.52E-02 |
| **FAM210B** | 0.854826757 | 0.939046225 | 0.695439642 | Downregulated | 4.91E-03 | 4.13E-02 |
| **SLC26A8** | 1.294510688 | 1.111706588 | 1.335111511 | Upregulated | 3.00E-05 | 5.32E-03 |
| **SLC18B1** | 0.758883546 | 0.944974293 | 0.894660117 | Downregulated | 1.48E-04 | 9.09E-03 |
| **LRG1** | 1.304600844 | 1.012782905 | 1.266657092 | Upregulated | 1.69E-03 | 2.45E-02 |
| **CLC** | 0.77816604 | 0.925657768 | 0.460056969 | Downregulated | 1.36E-02 | 6.96E-02 |
| **TDRD9** | 1.421413152 | 1.074108907 | 1.431687772 | Upregulated | 2.10E-04 | 1.05E-02 |
| **CCR7** | 0.929329209 | 0.946342055 | 0.649339494 | Downregulated | 1.63E-02 | 7.65E-02 |
| **ZNF792** | 0.925515272 | 0.960527575 | 0.750664752 | Downregulated | 3.56E-04 | 1.26E-02 |
| **TRABD2A** | 0.878122452 | 0.911710143 | 0.645965025 | Downregulated | 8.85E-04 | 1.86E-02 |
| **ZDHHC19** | 1.482948842 | 1.111942417 | 1.211732856 | Upregulated | 1.16E-05 | 5.16E-03 |
| **CPOX** | 0.768287857 | 0.993087668 | 0.817209352 | Downregulated | 8.98E-04 | 1.86E-02 |
| **SESN3** | 0.849954046 | 0.998839115 | 0.703118417 | Downregulated | 1.85E-02 | 8.18E-02 |
| **ZNF664** | 0.844374801 | 0.8707782 | 0.703405547 | Downregulated | 4.54E-05 | 6.23E-03 |
| **ZMAT2** | 0.881116902 | 0.968882716 | 0.768623342 | Downregulated | 3.72E-03 | 3.58E-02 |
| **ADRB2** | 0.757718736 | 0.959971022 | 1.146357972 | Downregulated | 1.21E-04 | 8.22E-03 |
| **CYP1B1** | 1.150878515 | 1.05960103 | 1.514388362 | Upregulated | 8.64E-03 | 5.52E-02 |
| **ANKRD46** | 0.756786894 | 0.940555876 | 0.90655443 | Downregulated | 1.34E-04 | 8.72E-03 |
| **CLEC12A** | 0.671641889 | 0.905953851 | 1.412198348 | Downregulated | 1.92E-02 | 8.34E-02 |
| **SLFN5** | 0.838883454 | 0.930406592 | 0.767612882 | Downregulated | 6.51E-05 | 6.34E-03 |
| **DCK** | 0.74310592 | 0.962568334 | 0.847437915 | Downregulated | 5.33E-05 | 6.23E-03 |
| **DYNC1I2** | 0.895510062 | 0.945856255 | 0.729082835 | Downregulated | 3.10E-04 | 1.19E-02 |
| **DPP4** | 0.906503094 | 0.939578805 | 0.7254311 | Downregulated | 7.60E-03 | 5.12E-02 |
| **LINC00189** | 1.10756045 | 1.204464993 | 1.451940588 | Upregulated | 1.06E-02 | 6.09E-02 |
| **SLC51A** | 1.260403681 | 0.988836067 | 1.307096013 | Upregulated | 8.70E-03 | 5.53E-02 |
| **EPB41** | 0.88588465 | 0.986692907 | 0.751580009 | Downregulated | 2.35E-02 | 9.30E-02 |
| **ETS2** | 1.119246675 | 1.039695351 | 1.370639933 | Upregulated | 6.05E-03 | 4.60E-02 |
| **EXT1** | 1.164941206 | 1.038146817 | 1.389171229 | Upregulated | 1.25E-03 | 2.15E-02 |
| **TBCEL** | 0.875699852 | 1.006791645 | 0.63037249 | Downregulated | 1.11E-02 | 6.24E-02 |
| **FCER1A** | 0.76937187 | 0.86149536 | 0.676932485 | Downregulated | 9.02E-04 | 1.86E-02 |
| **ZNF438** | 1.192663665 | 1.002521996 | 1.342305809 | Upregulated | 4.34E-03 | 3.88E-02 |
| **FKBP5** | 1.220567789 | 1.071593377 | 1.38394884 | Upregulated | 2.88E-03 | 3.15E-02 |
| **RPH3A** | 1.356185422 | 1.156957114 | 0.854184521 | Upregulated | 2.62E-04 | 1.12E-02 |
| **RPIA** | 0.766744277 | 0.989548505 | 0.694716644 | Downregulated | 1.15E-03 | 2.05E-02 |
| **GOLGA8A** | 0.760152449 | 0.957085628 | 0.789243704 | Downregulated | 2.25E-02 | 9.07E-02 |
| **PHLPP2** | 0.927526683 | 0.991915323 | 0.688208404 | Downregulated | 1.79E-02 | 8.05E-02 |
| **TNIK** | 0.969966759 | 0.963085435 | 0.730672162 | Downregulated | 2.24E-02 | 9.07E-02 |
| **ESYT1** | 0.910536179 | 0.946448437 | 0.743192405 | Downregulated | 7.53E-03 | 5.10E-02 |
| **ABCB10** | 0.691377125 | 1.004399678 | 0.78442025 | Downregulated | 2.51E-03 | 2.95E-02 |
| **MKRN1** | 0.856046776 | 1.017829261 | 0.723011699 | Downregulated | 1.11E-02 | 6.23E-02 |
| **SSBP3** | 0.890672252 | 0.946348084 | 0.753900667 | Downregulated | 9.89E-03 | 5.89E-02 |
| **LSM5** | 0.846697414 | 0.973491377 | 0.746216371 | Downregulated | 3.38E-03 | 3.43E-02 |
| **TMEM245** | 0.857322281 | 0.989977928 | 0.767844937 | Downregulated | 1.63E-02 | 7.65E-02 |
| **TXLNGY** | 1.039105884 | 1.033693281 | 0.738059213 | Upregulated | 2.20E-02 | 8.96E-02 |
| **ALPL** | 1.330166075 | 1.027573167 | 1.26639902 | Upregulated | 1.65E-03 | 2.41E-02 |
| **FUCA1** | 0.762236243 | 0.998777985 | 1.056141741 | Downregulated | 6.74E-03 | 4.83E-02 |
| **ZDHHC20** | 1.102486063 | 1.007862947 | 1.524970197 | Upregulated | 1.53E-02 | 7.40E-02 |
| **RALGAPA1** | 0.765712666 | 0.979185605 | 1.002087972 | Downregulated | 6.36E-03 | 4.68E-02 |
| **GABRR2** | 1.068059995 | 1.018487073 | 1.463149494 | Upregulated | 9.37E-03 | 5.74E-02 |
| **GCSAM** | 0.90299781 | 0.919499308 | 0.744600285 | Downregulated | 1.63E-04 | 9.38E-03 |
| **ASF1A** | 0.768677268 | 0.937855187 | 0.944194384 | Downregulated | 5.66E-05 | 6.23E-03 |
| **RWDD3** | 0.726429281 | 0.924571398 | 1.052495912 | Downregulated | 4.31E-06 | 3.76E-03 |
| **LDLRAP1** | 0.918783622 | 0.972789132 | 0.744106433 | Downregulated | 1.80E-02 | 8.08E-02 |
| **STAP1** | 0.75800479 | 0.854913573 | 0.998886728 | Downregulated | 1.38E-06 | 3.40E-03 |
| **AK5** | 0.963151193 | 0.868064396 | 0.650693633 | Downregulated | 2.66E-04 | 1.13E-02 |
| **GBP3** | 0.691976436 | 0.978629122 | 0.675845396 | Downregulated | 3.61E-03 | 3.52E-02 |
| **OFCC1** | 1.029290514 | 1.109005415 | 1.314855633 | Upregulated | 1.56E-02 | 7.45E-02 |
| **OPLAH** | 1.387704348 | 1.092262334 | 0.94917874 | Upregulated | 4.61E-06 | 3.80E-03 |
| **RANBP6** | 0.753924566 | 0.929572952 | 0.977331704 | Downregulated | 4.32E-04 | 1.38E-02 |
| **TSPAN13** | 0.750132128 | 0.903079761 | 0.973693384 | Downregulated | 5.76E-05 | 6.23E-03 |
| **SIGLEC9** | 1.113147993 | 1.018182577 | 1.41875008 | Upregulated | 9.48E-03 | 5.76E-02 |
| **GCLC** | 0.798059737 | 0.985140391 | 0.734541985 | Downregulated | 1.54E-02 | 7.42E-02 |
| **GPER1** | 1.038640733 | 1.057958155 | 1.331851087 | Upregulated | 9.55E-03 | 5.77E-02 |
| **HOXA-AS2** | 1.056041582 | 1.082140076 | 1.302489896 | Upregulated | 6.38E-03 | 4.69E-02 |
| **LSMEM1** | 1.052605943 | 1.056497161 | 1.429380059 | Upregulated | 2.28E-02 | 9.14E-02 |
| **TRAV5** | 0.96674744 | 0.873600816 | 0.754801519 | Downregulated | 2.64E-02 | 9.90E-02 |
| **TRAJ41** | 0.800184978 | 0.94274489 | 0.728602012 | Downregulated | 4.87E-03 | 4.11E-02 |
| **TRAC** | 0.814001407 | 0.968502792 | 0.756483953 | Downregulated | 3.34E-03 | 3.42E-02 |
| **GRB10** | 1.200182957 | 1.097125535 | 1.846575041 | Upregulated | 2.09E-03 | 2.69E-02 |
| **RGCC** | 0.773508279 | 1.040969553 | 0.684841425 | Downregulated | 2.62E-02 | 9.85E-02 |
| **N6AMT1** | 0.966010075 | 0.946806549 | 0.720159697 | Downregulated | 1.16E-03 | 2.06E-02 |
| **BLNK** | 0.7100668 | 0.916584984 | 1.019150461 | Downregulated | 9.09E-05 | 7.47E-03 |
| **GYG1** | 1.198463937 | 1.055498784 | 1.308620103 | Upregulated | 1.13E-03 | 2.04E-02 |
| **H1F0** | 0.778662767 | 0.944826551 | 0.75527309 | Downregulated | 1.45E-03 | 2.29E-02 |
| **HK3** | 1.193710534 | 1.043762579 | 1.337090657 | Upregulated | 2.43E-04 | 1.10E-02 |
| **HRH2** | 1.14335352 | 1.025327004 | 1.460784504 | Upregulated | 4.98E-03 | 4.15E-02 |
| **CLEC4D** | 1.244691844 | 1.059095754 | 1.376233636 | Upregulated | 1.25E-02 | 6.62E-02 |
| **ACADM** | 0.72294901 | 0.931705717 | 0.888004678 | Downregulated | 4.51E-05 | 6.23E-03 |
| **ZC3H12D** | 0.763520297 | 0.973482438 | 0.964746044 | Downregulated | 2.23E-03 | 2.78E-02 |
| **VSIG1** | 0.951689187 | 0.932907902 | 0.680110217 | Downregulated | 6.61E-03 | 4.78E-02 |
| **IL1R1** | 1.088237667 | 1.085324456 | 1.324171306 | Upregulated | 6.62E-03 | 4.78E-02 |
| **IL1RAP** | 1.163741033 | 1.016218763 | 1.310389926 | Upregulated | 1.70E-02 | 7.82E-02 |
| **ZBTB41** | 0.754891509 | 0.935102226 | 0.960192742 | Downregulated | 5.53E-04 | 1.51E-02 |
| **EIF3E** | 0.763281698 | 0.933355746 | 0.893291262 | Downregulated | 1.78E-04 | 9.55E-03 |
| **ITGA6** | 0.880921902 | 0.934155956 | 0.727050709 | Downregulated | 7.35E-04 | 1.72E-02 |
| **ITGA4** | 0.762726109 | 1.002603667 | 0.871636954 | Downregulated | 1.98E-02 | 8.48E-02 |
| **ITK** | 0.828031795 | 0.935888439 | 0.728740905 | Downregulated | 1.78E-03 | 2.50E-02 |
| **KCNMA1** | 1.060411635 | 1.013920425 | 1.330027995 | Upregulated | 2.55E-02 | 9.72E-02 |
| **THEMIS** | 0.775447702 | 0.957854233 | 0.716200293 | Downregulated | 3.65E-03 | 3.53E-02 |
| **LAMB2** | 1.05178463 | 1.022848768 | 1.305601216 | Upregulated | 4.10E-03 | 3.77E-02 |
| **LCK** | 0.895129932 | 0.936722088 | 0.74630463 | Downregulated | 5.76E-03 | 4.48E-02 |
| **LDHB** | 0.771738153 | 0.882836521 | 0.739359116 | Downregulated | 4.19E-07 | 1.45E-03 |
| **FAM102A** | 0.871206235 | 0.941970364 | 0.700723855 | Downregulated | 1.98E-03 | 2.62E-02 |
| **C8orf59** | 0.766848015 | 0.913278025 | 0.928728663 | Downregulated | 9.15E-04 | 1.86E-02 |
| **STS** | 1.022028895 | 1.064282589 | 1.399891154 | Upregulated | 1.18E-02 | 6.41E-02 |
| **MYH11** | 1.045219446 | 1.012559808 | 1.321052537 | Upregulated | 2.01E-02 | 8.54E-02 |
| **NDUFA5** | 0.687485039 | 0.994431573 | 0.965293271 | Downregulated | 1.38E-02 | 7.02E-02 |
| **NELL2** | 0.785324082 | 0.91373226 | 0.642718858 | Downregulated | 1.98E-04 | 1.02E-02 |
| **NFKBIA** | 1.092569214 | 1.021809105 | 1.352610917 | Upregulated | 2.27E-02 | 9.12E-02 |
| **NPAT** | 0.914469682 | 0.944889105 | 0.746505649 | Downregulated | 4.83E-05 | 6.23E-03 |
| **NUCB2** | 0.749613912 | 0.965573738 | 0.800992598 | Downregulated | 6.57E-04 | 1.65E-02 |
| **ODC1** | 0.845725099 | 0.979414903 | 0.678777203 | Downregulated | 2.13E-03 | 2.72E-02 |
| **TRAT1** | 0.733528042 | 0.899100503 | 0.82481524 | Downregulated | 4.26E-04 | 1.37E-02 |
| **HEBP1** | 0.758344883 | 0.942046862 | 0.997593437 | Downregulated | 1.95E-02 | 8.42E-02 |
| **MTERF3** | 0.725027777 | 1.009714238 | 0.784870593 | Downregulated | 1.05E-03 | 1.99E-02 |
| **ISOC1** | 0.744102291 | 0.959780828 | 0.900515365 | Downregulated | 5.40E-05 | 6.23E-03 |
| **IER3IP1** | 0.747273253 | 0.935152525 | 1.048655375 | Downregulated | 1.33E-03 | 2.20E-02 |
| **LEF1** | 0.89312532 | 0.94810432 | 0.648205418 | Downregulated | 4.10E-03 | 3.77E-02 |
| **RWDD1** | 0.734226044 | 0.970599269 | 0.925852181 | Downregulated | 7.11E-04 | 1.69E-02 |
| **TMEM14C** | 0.766956638 | 0.961470122 | 0.996266281 | Downregulated | 6.50E-04 | 1.64E-02 |
| **C9orf78** | 0.747426025 | 0.943116915 | 0.835641341 | Downregulated | 8.20E-04 | 1.79E-02 |
| **ACP1** | 0.827939727 | 0.998587402 | 0.740735364 | Downregulated | 2.11E-02 | 8.78E-02 |
| **PFKFB2** | 1.158034602 | 1.061792048 | 1.541465818 | Upregulated | 1.62E-02 | 7.61E-02 |
| **SERPINI1** | 0.920203336 | 0.872420873 | 0.755891687 | Downregulated | 1.81E-04 | 9.60E-03 |
| **PMAIP1** | 0.763614023 | 0.90756935 | 0.942915282 | Downregulated | 2.69E-05 | 5.29E-03 |
| **GPR84** | 1.309922831 | 1.045083064 | 1.230285771 | Upregulated | 1.01E-02 | 5.94E-02 |
| **SSH1** | 1.103763322 | 1.011710802 | 1.301471489 | Upregulated | 1.05E-02 | 6.06E-02 |
| **LRRN3** | 0.746608929 | 0.851990839 | 0.635064055 | Downregulated | 1.61E-04 | 9.38E-03 |
| **TRMT61B** | 0.748225902 | 0.917357416 | 0.965068688 | Downregulated | 3.55E-05 | 5.92E-03 |
| **PCMTD2** | 0.765062041 | 0.995288447 | 0.915165409 | Downregulated | 1.69E-03 | 2.45E-02 |
| **TBC1D19** | 0.975242013 | 0.920327362 | 0.765558945 | Downregulated | 7.28E-03 | 5.04E-02 |
| **CCDC91** | 0.736070719 | 0.93416798 | 0.810794476 | Downregulated | 4.66E-05 | 6.23E-03 |
| **DNAJA4** | 0.861492768 | 0.962857691 | 0.693398427 | Downregulated | 1.37E-02 | 7.00E-02 |
| **LRRC40** | 0.748284839 | 0.942711517 | 1.013224764 | Downregulated | 9.18E-04 | 1.87E-02 |
| **ZNF83** | 0.746961917 | 0.88726961 | 0.966440427 | Downregulated | 1.95E-04 | 1.01E-02 |
| **MBNL3** | 0.827957967 | 0.998240344 | 0.665773915 | Downregulated | 2.31E-02 | 9.23E-02 |
| **GSDMB** | 0.896909696 | 0.937753577 | 0.750075282 | Downregulated | 1.64E-03 | 2.41E-02 |
| **CMAS** | 0.756964373 | 0.962533005 | 0.881010378 | Downregulated | 1.60E-03 | 2.39E-02 |
| **BAIAP2L1** | 1.055366255 | 1.003667984 | 1.324191935 | Upregulated | 7.74E-03 | 5.18E-02 |
| **ANKH** | 0.871200048 | 0.951165421 | 0.708840165 | Downregulated | 4.07E-03 | 3.75E-02 |
| **PITHD1** | 0.810955459 | 1.017152806 | 0.642427149 | Downregulated | 2.18E-02 | 8.93E-02 |
| **CD177** | 1.9396034 | 1.374892682 | 0.942545513 | Upregulated | 3.95E-05 | 6.01E-03 |
| **NLRC4** | 1.204062926 | 1.08405266 | 1.307344198 | Upregulated | 5.38E-04 | 1.50E-02 |
| **ZNF77** | 0.979497885 | 0.968645496 | 0.738605331 | Downregulated | 2.55E-02 | 9.71E-02 |
| **RAN** | 0.765738666 | 0.956617262 | 0.984950614 | Downregulated | 6.70E-04 | 1.67E-02 |
| **PLEKHA1** | 0.847367513 | 0.948450287 | 0.74399352 | Downregulated | 6.60E-04 | 1.65E-02 |
| **ABCE1** | 0.740999998 | 0.93357816 | 0.817176113 | Downregulated | 2.55E-04 | 1.12E-02 |
| **RPS27A** | 0.876137359 | 0.972921782 | 0.766569766 | Downregulated | 5.76E-03 | 4.48E-02 |
| **SATB1** | 0.901234446 | 0.98066785 | 0.759767283 | Downregulated | 6.65E-03 | 4.80E-02 |
| **SCML1** | 0.875514108 | 0.929442804 | 0.704127029 | Downregulated | 7.63E-04 | 1.74E-02 |
| **NECAB1** | 1.042771094 | 1.216461937 | 1.521971958 | Upregulated | 5.37E-05 | 6.23E-03 |
| **SMIM15** | 0.768781737 | 0.970442676 | 1.268798343 | Downregulated | 1.37E-02 | 7.00E-02 |
| **C11orf1** | 0.880032817 | 0.960358307 | 0.762735413 | Downregulated | 3.20E-03 | 3.35E-02 |
| **SLC1A3** | 1.090061472 | 1.112104817 | 1.418643878 | Upregulated | 6.19E-05 | 6.23E-03 |
| **SLC8A1** | 1.03847497 | 1.106156407 | 1.426259702 | Upregulated | 1.23E-03 | 2.13E-02 |
| **SPI1** | 1.30051331 | 1.007050737 | 1.077169548 | Upregulated | 6.26E-03 | 4.65E-02 |
| **BPGM** | 0.767308303 | 0.970785686 | 0.749730817 | Downregulated | 1.96E-02 | 8.43E-02 |
| **SSB** | 0.737961866 | 0.915909065 | 0.989706805 | Downregulated | 2.14E-05 | 5.27E-03 |
| **TCF7** | 0.866595392 | 0.941871495 | 0.684761663 | Downregulated | 7.17E-03 | 4.98E-02 |
| **PRDX2** | 0.821699794 | 0.975509068 | 0.714157053 | Downregulated | 1.79E-02 | 8.04E-02 |
| **TFDP2** | 0.915301674 | 0.972490334 | 0.625641203 | Downregulated | 9.65E-03 | 5.80E-02 |
| **TFPI** | 1.044097212 | 1.117168095 | 0.750437685 | Upregulated | 9.33E-03 | 5.74E-02 |
| **TLR5** | 1.210743569 | 1.032898725 | 1.389898046 | Upregulated | 9.52E-04 | 1.89E-02 |
| **LINC02363** | 1.071383847 | 1.054741571 | 1.366852097 | Upregulated | 2.24E-04 | 1.09E-02 |
| **TXK** | 0.843305954 | 0.940617661 | 0.693931009 | Downregulated | 1.53E-03 | 2.33E-02 |
| **ZKSCAN8** | 0.950410573 | 0.981322414 | 0.762548263 | Downregulated | 9.45E-03 | 5.76E-02 |
| **IL1R2** | 1.231921476 | 1.051452709 | 1.625438109 | Upregulated | 4.77E-03 | 4.06E-02 |
| **ATP13A3** | 1.088491377 | 1.076928736 | 1.358306685 | Upregulated | 2.21E-03 | 2.77E-02 |
| **GALNT14** | 1.460989585 | 1.074437781 | 1.196744541 | Upregulated | 1.33E-05 | 5.16E-03 |
| **NSUN7** | 1.326745857 | 1.082550239 | 1.687953125 | Upregulated | 4.51E-04 | 1.39E-02 |
| **ZFAND1** | 0.760347216 | 0.925127866 | 0.970354626 | Downregulated | 9.99E-04 | 1.94E-02 |
| **CAMKMT** | 0.990017075 | 0.967332629 | 0.760061446 | Downregulated | 2.31E-02 | 9.23E-02 |
| **ATF7IP2** | 0.925777861 | 0.97781423 | 0.754859984 | Downregulated | 1.52E-02 | 7.38E-02 |
| **CAMK4** | 0.842826394 | 0.9242124 | 0.63792032 | Downregulated | 1.70E-04 | 9.39E-03 |
| **ISCA1** | 0.768360703 | 0.957777153 | 0.734876633 | Downregulated | 3.40E-03 | 3.44E-02 |
| **CASP5** | 1.235909875 | 1.069886171 | 1.647471585 | Upregulated | 2.52E-03 | 2.95E-02 |
| **TMTC1** | 1.082879352 | 1.432527616 | 1.830927476 | Upregulated | 1.30E-02 | 6.78E-02 |
| **EIF2A** | 0.759180482 | 0.945306318 | 0.874167798 | Downregulated | 5.93E-05 | 6.23E-03 |
| **DCTN5** | 0.946991051 | 0.962816937 | 0.736392705 | Downregulated | 3.26E-03 | 3.37E-02 |
| **GAS7** | 1.183923072 | 1.053219547 | 1.470584972 | Upregulated | 1.75E-03 | 2.49E-02 |
| **CST7** | 1.188969828 | 1.033458464 | 1.334064851 | Upregulated | 3.50E-03 | 3.49E-02 |
| **MKNK1** | 1.195770453 | 1.000998202 | 1.3436189 | Upregulated | 9.88E-03 | 5.88E-02 |
| **CBLB** | 0.880182579 | 0.97116285 | 0.768116139 | Downregulated | 8.83E-03 | 5.58E-02 |
| **SUCLA2** | 0.743568141 | 0.926776389 | 1.070324134 | Downregulated | 9.77E-04 | 1.91E-02 |
| **INPP4B** | 0.733271201 | 0.957170872 | 0.886319517 | Downregulated | 2.27E-03 | 2.80E-02 |
| **CCNC** | 0.750028549 | 0.945681523 | 1.07372355 | Downregulated | 3.61E-03 | 3.52E-02 |
| **CCND2** | 0.971320128 | 0.928991876 | 0.74602795 | Downregulated | 4.22E-03 | 3.83E-02 |
| **LHX4** | 1.088305428 | 0.996304008 | 1.443376411 | Upregulated | 2.29E-03 | 2.81E-02 |
| **SELENBP1** | 0.872312469 | 0.973544289 | 0.615836275 | Downregulated | 2.54E-02 | 9.68E-02 |
| **UBE3D** | 0.933572703 | 0.974887918 | 0.750812626 | Downregulated | 8.56E-04 | 1.83E-02 |
| **SOCS3** | 1.291986831 | 1.026712453 | 1.464462174 | Upregulated | 9.61E-04 | 1.90E-02 |
| **TCEAL8** | 0.755431913 | 0.963990762 | 0.894451746 | Downregulated | 2.55E-04 | 1.12E-02 |
| **CD3D** | 0.840081835 | 0.924571411 | 0.74780274 | Downregulated | 4.66E-03 | 4.03E-02 |
| **EBAG9** | 0.758356008 | 0.914525878 | 0.994021007 | Downregulated | 3.31E-07 | 1.45E-03 |
| **CD3G** | 0.924132374 | 0.869000105 | 0.744139082 | Downregulated | 4.73E-04 | 1.42E-02 |
| **OXNAD1** | 0.861317929 | 0.932752003 | 0.757184996 | Downregulated | 5.21E-04 | 1.49E-02 |
| **TMEM88** | 1.149292668 | 1.024886979 | 1.358550719 | Upregulated | 1.93E-03 | 2.59E-02 |
| **METTL18** | 0.763100638 | 0.89849737 | 0.931876155 | Downregulated | 2.17E-05 | 5.27E-03 |
| **CD8B** | 0.856761369 | 0.909815767 | 0.742642806 | Downregulated | 6.03E-03 | 4.59E-02 |
| **UBE2Q2** | 0.764893799 | 0.958358597 | 0.842903284 | Downregulated | 8.68E-04 | 1.84E-02 |
| **MS4A1** | 0.716716515 | 0.912668821 | 1.099021075 | Downregulated | 6.83E-04 | 1.68E-02 |
| **HMGN3** | 0.750636887 | 0.937432463 | 0.998647905 | Downregulated | 3.68E-04 | 1.27E-02 |
| **MGAM2** | 1.033862323 | 1.111627409 | 1.461101492 | Upregulated | 5.03E-03 | 4.17E-02 |
| **CD28** | 0.913342929 | 0.93322715 | 0.720139892 | Downregulated | 1.11E-02 | 6.23E-02 |
| **RAB3D** | 1.127020624 | 1.008471777 | 1.393555158 | Upregulated | 7.78E-03 | 5.20E-02 |
| **PDE4DIP** | 0.911962588 | 0.964099788 | 0.739780582 | Downregulated | 6.60E-03 | 4.78E-02 |
| **RNF144A** | 0.852663609 | 1.031726685 | 0.696587496 | Downregulated | 2.02E-02 | 8.57E-02 |
| **TESPA1** | 0.8783266 | 0.967516917 | 0.719321928 | Downregulated | 1.09E-02 | 6.17E-02 |
| **CASP8AP2** | 0.7614337 | 0.922072218 | 0.884480923 | Downregulated | 5.95E-05 | 6.23E-03 |

**Suppl. Table 1. Significant genes from cross-study meta-analysis.** FC_GSE46510, fold-change calculated using GSE46510 samples; FC_GSE59491, fold-change calculated using GSE59491 samples; FC_GSE73685, fold-change calculated using GSE73685 samples; adj.P.Val, adjusted p-value.
