# Supplementary material for: Meta-Analysis of Maternal and Fetal Transcriptomic Data Elucidates the Role of Adaptive and Innate Immunity in Preterm Birth
Source: Front Immunol. 2018 May 9;9:993. doi: 10.3389/fimmu.2018.00993 (PMC5954243; doi:10.3389/fimmu.2018.00993)
Supplement: Supplementary file 2 [file Table_2.docx]

| **Pathway ID** | **Pathway description** | **Observed gene count** | **False discovery rate** |
| --- | --- | --- | --- |
| GO.0045321 | leukocyte activation | 18 | 1.36E-06 |
| GO.0046649 | lymphocyte activation | 16 | 3.06E-06 |
| GO.0007159 | leukocyte cell-cell adhesion | 14 | 7.20E-06 |
| GO.0042110 | T cell activation | 13 | 7.20E-06 |
| GO.0050851 | antigen receptor-mediated signaling pathway | 10 | 8.45E-06 |
| GO.0050854 | regulation of antigen receptor-mediated signaling pathway | 7 | 8.87E-06 |
| GO.0050852 | T cell receptor signaling pathway | 9 | 1.05E-05 |
| GO.0050857 | positive regulation of antigen receptor-mediated signaling pathway | 5 | 3.58E-05 |
| GO.0050862 | positive regulation of T cell receptor signaling pathway | 4 | 8.13E-05 |
| GO.0030098 | lymphocyte differentiation | 11 | 0.000101 |
| GO.0001775 | cell activation | 18 | 0.000121 |
| GO.0030217 | T cell differentiation | 9 | 0.000136 |
| GO.0022407 | regulation of cell-cell adhesion | 13 | 0.000284 |
| GO.0002521 | leukocyte differentiation | 12 | 0.000345 |
| GO.0050856 | regulation of T cell receptor signaling pathway | 5 | 0.000739 |
| GO.0002682 | regulation of immune system process | 25 | 0.000864 |
| GO.0050865 | regulation of cell activation | 13 | 0.00262 |
| GO.0030155 | regulation of cell adhesion | 15 | 0.00287 |
| GO.1903037 | regulation of leukocyte cell-cell adhesion | 10 | 0.00417 |
| GO.0034110 | regulation of homotypic cell-cell adhesion | 10 | 0.00533 |
| GO.0002694 | regulation of leukocyte activation | 12 | 0.00539 |
| GO.0002376 | immune system process | 29 | 0.00712 |
| GO.0050778 | positive regulation of immune response | 14 | 0.00712 |
| GO.0050776 | regulation of immune response | 17 | 0.00742 |
| GO.0002863 | positive regulation of inflammatory response to antigenic stimulus | 3 | 0.0077 |
| GO.0002684 | positive regulation of immune system process | 17 | 0.00857 |
| GO.0030097 | hemopoiesis | 13 | 0.013 |
| GO.0050863 | regulation of T cell activation | 9 | 0.0152 |
| GO.0002768 | immune response-regulating cell surface receptor signaling pathway | 11 | 0.0164 |
| GO.0031295 | T cell costimulation | 5 | 0.0202 |
| GO.0022409 | positive regulation of cell-cell adhesion | 8 | 0.0208 |
| GO.0045058 | T cell selection | 4 | 0.0208 |
| GO.0043383 | negative T cell selection | 3 | 0.0226 |
| GO.0051249 | regulation of lymphocyte activation | 10 | 0.0249 |
| GO.0007167 | enzyme linked receptor protein signaling pathway | 17 | 0.0284 |
| GO.0002253 | activation of immune response | 11 | 0.0352 |
| GO.0002520 | immune system development | 13 | 0.0373 |
| GO.0038093 | Fc receptor signaling pathway | 8 | 0.0442 |
| GO.0038095 | Fc-epsilon receptor signaling pathway | 7 | 0.0456 |

**Suppl. Table 2. Gene Ontology (GO) biological processes enriched by downregulated genes from cross-study meta-analysis.** Observed gene count, number of significant genes included in given pathways.
