# Supplementary material for: Meta-Analysis of Maternal and Fetal Transcriptomic Data Elucidates the Role of Adaptive and Innate Immunity in Preterm Birth
Source: Front Immunol. 2018 May 9;9:993. doi: 10.3389/fimmu.2018.00993 (PMC5954243; doi:10.3389/fimmu.2018.00993)
Supplement: Supplementary file 3 [file Table_3.docx]

| **Genes** | **FC_GSE59491** | **Directionality** | **P.Value** | **adj.P.Val** |
| --- | --- | --- | --- | --- |
| **ANKRD46** | 0.9259474 | Downregulated | 6.26E-03 | 2.67E-01 |
| **ATP9A** | 1.155575287 | Upregulated | 8.12E-03 | 2.82E-01 |
| **CBLB** | 0.930859678 | Downregulated | 3.43E-03 | 2.35E-01 |
| **CCND2** | 0.912609557 | Downregulated | 9.96E-04 | 1.85E-01 |
| **CD177** | 1.485407845 | Upregulated | 2.61E-03 | 2.30E-01 |
| **CD3G** | 0.896812728 | Downregulated | 2.81E-03 | 2.33E-01 |
| **DYNC1I2** | 0.925340697 | Downregulated | 3.03E-03 | 2.35E-01 |
| **ESYT1** | 0.921494094 | Downregulated | 6.57E-03 | 2.75E-01 |
| **GRB10** | 1.155601245 | Upregulated | 7.23E-04 | 1.70E-01 |
| **IL1R1** | 1.150958552 | Upregulated | 3.96E-03 | 2.45E-01 |
| **LCK** | 0.91540983 | Downregulated | 3.78E-03 | 2.39E-01 |
| **LDHB** | 0.9017703 | Downregulated | 1.57E-03 | 2.08E-01 |
| **LINC02363** | 1.112635812 | Upregulated | 7.06E-03 | 2.78E-01 |
| **NECAB1** | 1.178366975 | Upregulated | 2.41E-03 | 2.30E-01 |
| **NLRC4** | 1.116708767 | Upregulated | 6.57E-03 | 2.75E-01 |
| **SLFN5** | 0.905549702 | Downregulated | 5.34E-04 | 1.63E-01 |
| **TFPI** | 1.119179494 | Upregulated | 7.65E-03 | 2.80E-01 |
| **TNIK** | 0.926242722 | Downregulated | 4.83E-03 | 2.51E-01 |

**Suppl. Table 3. Significant genes from ad-hoc T2 analysis.** FC_GSE59491, fold-change calculated using GSE59491 T2 samples; adj.P.Val, adjusted p-value.
