# Supplementary material for: Meta-Analysis of Maternal and Fetal Transcriptomic Data Elucidates the Role of Adaptive and Innate Immunity in Preterm Birth
Source: Front Immunol. 2018 May 9;9:993. doi: 10.3389/fimmu.2018.00993 (PMC5954243; doi:10.3389/fimmu.2018.00993)
Supplement: Supplementary file 4 [file Table_4.docx]

| **Gene** | **P-value** | **Log FC** | **P-value** | **Log FC** |
| --- | --- | --- | --- | --- |
|  | **(meta-analysis)** | **(meta-analysis)** | **(T2)** | **(T2)** |
| **ANKRD46** | 1.34E-04 | 0.7568 | 6.26E-03 | 0.9259 |
| **ATP9A** | 9.79E-04 | 1.3009 | 8.12E-03 | 1.1556 |
| **CBLB** | 8.83E-03 | 0.7681 | 3.43E-03 | 0.9309 |
| **CCND2** | 4.22E-03 | 0.7460 | 9.96E-04 | 0.9126 |
| **CD177*** | 3.95E-05 | 1.3749 | 2.61E-03 | 1.4854 |
|  |  | 1.9396 |  |  |
| **CD3G** | 4.73E-04 | 0.7441 | 2.81E-03 | 0.8968 |
| **DYNC1I2** | 3.10E-04 | 0.7291 | 3.03E-03 | 0.9253 |
| **ESYT1** | 7.53E-03 | 0.7432 | 6.57E-03 | 0.9215 |
| **GRB10** | 2.09E-03 | 1.8466 | 7.23E-04 | 1.1556 |
| **IL1R1** | 6.62E-03 | 1.3242 | 3.96E-03 | 1.1510 |
| **LCK** | 5.76E-03 | 0.7463 | 3.78E-03 | 0.9154 |
| **LDHB** | 4.19E-07 | 0.7394 | 1.57E-03 | 0.9018 |
| **LINC02363** | 2.24E-04 | 1.3669 | 7.06E-03 | 1.1126 |
| **NECAB1** | 5.37E-05 | 1.5220 | 2.41E-03 | 1.1784 |
| **NLRC4** | 5.38E-04 | 1.3073 | 6.57E-03 | 1.1167 |
| **SLFN5** | 6.51E-05 | 0.7676 | 5.34E-04 | 0.9055 |
| **TFPI** | 9.33E-03 | 0.7504 | 7.65E-03 | 1.1192 |
| **TNIK** | 2.24E-02 | 0.7307 | 4.83E-03 | 0.9262 |

**Suppl. Table 4. Comparison of p-value and fold change for overlapping significant genes from T2 analysis and meta-analysis.** Log FC (meta-analysis) reported is for the study in which the gene was found to be significant. Genes with * were found to be significant in two individual studies (GSE46561 & GSE59491) and thus fold-change calculations from both studies are included. Log FC, log fold-change.
