# Supplementary material for: Meta-Analysis of Maternal and Fetal Transcriptomic Data Elucidates the Role of Adaptive and Innate Immunity in Preterm Birth
Source: Front Immunol. 2018 May 9;9:993. doi: 10.3389/fimmu.2018.00993 (PMC5954243; doi:10.3389/fimmu.2018.00993)
Supplement: Supplementary file 5 [file Table_5.docx]

| **Genes** | **FC_GSE73685** | **Directionality** | **P.Value** | **adj.P.Val** |
| --- | --- | --- | --- | --- |
| **PI3** | 0.147982228 | Downregulated | 1.09E-08 | 2.25E-04 |
| **HBD** | 0.256928238 | Downregulated | 6.78E-08 | 7.00E-04 |
| **CALR** | 1.503963116 | Upregulated | 2.33E-07 | 1.61E-03 |
| **LINC00999** | 0.68049323 | Downregulated | 5.21E-07 | 2.69E-03 |
| **CXCR1** | 0.364494145 | Downregulated | 1.31E-06 | 5.41E-03 |
| **CRKL** | 1.474261419 | Upregulated | 1.71E-06 | 5.88E-03 |
| **HCAR3** | 0.308895133 | Downregulated | 2.43E-06 | 6.35E-03 |
| **TMEM252** | 0.588722515 | Downregulated | 2.46E-06 | 6.35E-03 |
| **CSF2RB** | 0.554199987 | Downregulated | 3.57E-06 | 7.32E-03 |
| **MRPL14** | 1.747080461 | Upregulated | 4.09E-06 | 7.32E-03 |
| **RNF19B** | 0.596239673 | Downregulated | 4.25E-06 | 7.32E-03 |
| **QPCT** | 0.391870389 | Downregulated | 5.33E-06 | 7.84E-03 |
| **CDC42EP2** | 0.647122793 | Downregulated | 5.62E-06 | 7.84E-03 |
| **H19** | 6.776214775 | Upregulated | 5.69E-06 | 7.84E-03 |
| **CYP4F3** | 0.498981015 | Downregulated | 6.30E-06 | 7.98E-03 |
| **FRAT2** | 0.622204508 | Downregulated | 6.57E-06 | 7.98E-03 |
| **CXCR2** | 0.419281148 | Downregulated | 8.03E-06 | 9.22E-03 |
| **NADK** | 0.629492574 | Downregulated | 8.92E-06 | 9.71E-03 |
| **PLXNC1** | 0.430280029 | Downregulated | 9.98E-06 | 9.91E-03 |
| **SOD2** | 0.531728948 | Downregulated | 1.10E-05 | 9.91E-03 |
| **ZDHHC18** | 0.51359278 | Downregulated | 1.14E-05 | 9.91E-03 |
| **HBE1** | 8.325561707 | Upregulated | 1.18E-05 | 9.91E-03 |
| **AARS** | 1.491907883 | Upregulated | 1.19E-05 | 9.91E-03 |
| **HCG27** | 0.466523653 | Downregulated | 1.24E-05 | 9.91E-03 |
| **NET1** | 2.203709531 | Upregulated | 1.25E-05 | 9.91E-03 |
| **FFAR2** | 0.387256617 | Downregulated | 1.48E-05 | 1.11E-02 |
| **LITAF** | 0.604608175 | Downregulated | 1.55E-05 | 1.11E-02 |
| **OSGIN2** | 0.584902206 | Downregulated | 1.57E-05 | 1.11E-02 |
| **EPHB1** | 0.673483329 | Downregulated | 1.63E-05 | 1.11E-02 |
| **NCF2** | 0.44307106 | Downregulated | 1.72E-05 | 1.11E-02 |
| **SECTM1** | 0.502608566 | Downregulated | 1.78E-05 | 1.11E-02 |
| **NDUFA6** | 1.526316241 | Upregulated | 1.85E-05 | 1.11E-02 |
| **CD300LF** | 0.632510028 | Downregulated | 1.98E-05 | 1.11E-02 |
| **IL13RA1** | 0.43467984 | Downregulated | 1.99E-05 | 1.11E-02 |
| **MIR223** | 0.45074236 | Downregulated | 2.16E-05 | 1.11E-02 |
| **EHD3** | 1.406040999 | Upregulated | 2.21E-05 | 1.11E-02 |
| **IL6ST** | 1.534764344 | Upregulated | 2.30E-05 | 1.11E-02 |
| **TMEM55A** | 0.457842573 | Downregulated | 2.35E-05 | 1.11E-02 |
| **GLT1D1** | 0.406926977 | Downregulated | 2.37E-05 | 1.11E-02 |
| **LYST** | 0.529076891 | Downregulated | 2.40E-05 | 1.11E-02 |
| **LARP1** | 1.916221804 | Upregulated | 2.45E-05 | 1.11E-02 |
| **TMEM154** | 0.426905523 | Downregulated | 2.46E-05 | 1.11E-02 |
| **HSPC102** | 0.358994249 | Downregulated | 2.49E-05 | 1.11E-02 |
| **LINC00528** | 0.68525867 | Downregulated | 2.55E-05 | 1.11E-02 |
| **IL1B** | 0.377711301 | Downregulated | 2.62E-05 | 1.11E-02 |
| **CRISP3** | 0.160141292 | Downregulated | 2.65E-05 | 1.11E-02 |
| **CEACAM3** | 0.530135917 | Downregulated | 2.67E-05 | 1.11E-02 |
| **IPMK** | 0.577832327 | Downregulated | 2.70E-05 | 1.11E-02 |
| **PELI1** | 0.453896572 | Downregulated | 2.79E-05 | 1.11E-02 |
| **NAMPTP1** | 0.341806943 | Downregulated | 2.82E-05 | 1.11E-02 |
| **POLE3** | 1.330136052 | Upregulated | 2.86E-05 | 1.11E-02 |
| **DYNLT1** | 0.613154804 | Downregulated | 2.86E-05 | 1.11E-02 |
| **HSPA9** | 1.475436018 | Upregulated | 2.94E-05 | 1.11E-02 |
| **SLC35E1** | 1.625869518 | Upregulated | 2.94E-05 | 1.11E-02 |
| **MBP** | 0.74579852 | Downregulated | 3.06E-05 | 1.13E-02 |
| **SND1** | 1.314331029 | Upregulated | 3.14E-05 | 1.14E-02 |
| **SLC15A4** | 0.578873598 | Downregulated | 3.19E-05 | 1.14E-02 |
| **PNMA1** | 1.377004166 | Upregulated | 3.27E-05 | 1.14E-02 |
| **LRRC25** | 0.623806976 | Downregulated | 3.42E-05 | 1.17E-02 |
| **TMEM135** | 1.537720361 | Upregulated | 3.46E-05 | 1.17E-02 |
| **LRRC6** | 0.499421249 | Downregulated | 3.54E-05 | 1.17E-02 |
| **MXD1** | 0.521399826 | Downregulated | 3.58E-05 | 1.17E-02 |
| **RUNDC3A** | 2.730651398 | Upregulated | 3.65E-05 | 1.17E-02 |
| **PLBD1** | 0.450271124 | Downregulated | 3.70E-05 | 1.17E-02 |
| **KCNJ15** | 0.308455901 | Downregulated | 3.72E-05 | 1.17E-02 |
| **AQP9** | 0.386018599 | Downregulated | 3.86E-05 | 1.17E-02 |
| **RNF24** | 0.575283651 | Downregulated | 3.88E-05 | 1.17E-02 |
| **IFNGR2** | 0.628318533 | Downregulated | 3.89E-05 | 1.17E-02 |
| **HMGA1P1** | 1.358884892 | Upregulated | 4.05E-05 | 1.17E-02 |
| **MAGED2** | 1.562935356 | Upregulated | 4.07E-05 | 1.17E-02 |
| **SSSCA1** | 1.641832354 | Upregulated | 4.26E-05 | 1.21E-02 |
| **FBXL5** | 0.527180826 | Downregulated | 4.46E-05 | 1.24E-02 |
| **MAGED1** | 1.529416771 | Upregulated | 4.61E-05 | 1.26E-02 |
| **C5AR2** | 0.544585547 | Downregulated | 4.69E-05 | 1.26E-02 |
| **NRBF2** | 0.572220276 | Downregulated | 4.74E-05 | 1.26E-02 |
| **RNF149** | 0.512926289 | Downregulated | 4.82E-05 | 1.26E-02 |
| **IRF1** | 0.639532824 | Downregulated | 4.85E-05 | 1.26E-02 |
| **BASP1** | 0.497768358 | Downregulated | 4.91E-05 | 1.26E-02 |
| **LYN** | 0.47557254 | Downregulated | 4.92E-05 | 1.26E-02 |
| **TNFRSF10C** | 0.457093268 | Downregulated | 5.07E-05 | 1.28E-02 |
| **PNKD** | 2.345597106 | Upregulated | 5.25E-05 | 1.30E-02 |
| **SUSD6** | 0.709095274 | Downregulated | 5.27E-05 | 1.30E-02 |
| **KCNJ2** | 0.421569525 | Downregulated | 5.42E-05 | 1.32E-02 |
| **LRRC4** | 0.539356039 | Downregulated | 5.53E-05 | 1.33E-02 |
| **EGR1** | 1.707008761 | Upregulated | 5.60E-05 | 1.33E-02 |
| **EIF4G1** | 1.53572217 | Upregulated | 5.74E-05 | 1.35E-02 |
| **UBE2D1** | 0.504313608 | Downregulated | 5.88E-05 | 1.37E-02 |
| **DNMT1** | 1.403650819 | Upregulated | 6.18E-05 | 1.42E-02 |
| **RPL41** | 1.673454316 | Upregulated | 6.48E-05 | 1.46E-02 |
| **CDCA7** | 1.567482622 | Upregulated | 6.81E-05 | 1.51E-02 |
| **CHST15** | 0.553276886 | Downregulated | 6.89E-05 | 1.51E-02 |
| **FAM129A** | 0.413852773 | Downregulated | 7.06E-05 | 1.51E-02 |
| **PROK2** | 0.416070658 | Downregulated | 7.10E-05 | 1.51E-02 |
| **ADI1** | 1.363205598 | Upregulated | 7.11E-05 | 1.51E-02 |
| **CYTH4** | 0.590527424 | Downregulated | 7.30E-05 | 1.54E-02 |
| **TNFSF13B** | 0.485673736 | Downregulated | 7.52E-05 | 1.57E-02 |
| **CTBS** | 0.517491087 | Downregulated | 7.61E-05 | 1.57E-02 |
| **CASP5** | 0.356887266 | Downregulated | 7.81E-05 | 1.59E-02 |
| **C9orf66** | 0.570305146 | Downregulated | 7.88E-05 | 1.59E-02 |
| **MEFV** | 0.582316971 | Downregulated | 7.96E-05 | 1.59E-02 |
| **TMEM71** | 0.54243664 | Downregulated | 7.99E-05 | 1.59E-02 |
| **SF3B3** | 1.522082266 | Upregulated | 8.15E-05 | 1.59E-02 |
| **PPP1R12B** | 0.648919656 | Downregulated | 8.16E-05 | 1.59E-02 |
| **FAS** | 0.545013109 | Downregulated | 8.63E-05 | 1.64E-02 |
| **HIF3A** | 2.704892194 | Upregulated | 8.65E-05 | 1.64E-02 |
| **MLLT3** | 1.501847559 | Upregulated | 8.66E-05 | 1.64E-02 |
| **F2RL1** | 0.620799795 | Downregulated | 8.70E-05 | 1.64E-02 |
| **GCA** | 0.485829889 | Downregulated | 8.79E-05 | 1.64E-02 |
| **CSF3R** | 0.522136357 | Downregulated | 8.98E-05 | 1.65E-02 |
| **GOT1** | 1.494705145 | Upregulated | 9.03E-05 | 1.65E-02 |
| **EFHD2** | 0.663824701 | Downregulated | 9.10E-05 | 1.65E-02 |
| **ADAMTSL4-AS1** | 0.464529699 | Downregulated | 9.25E-05 | 1.66E-02 |
| **BZW2** | 1.772600094 | Upregulated | 9.44E-05 | 1.68E-02 |
| **MPC2** | 1.675037286 | Upregulated | 9.58E-05 | 1.69E-02 |
| **FAM49B** | 0.599694937 | Downregulated | 9.97E-05 | 1.74E-02 |
| **REPS2** | 0.548191827 | Downregulated | 1.00E-04 | 1.74E-02 |
| **S100A9** | 0.589050352 | Downregulated | 1.04E-04 | 1.79E-02 |
| **OGFRL1** | 0.543521968 | Downregulated | 1.06E-04 | 1.82E-02 |
| **CDK4** | 1.510386839 | Upregulated | 1.08E-04 | 1.82E-02 |
| **SPOPL** | 0.526730843 | Downregulated | 1.09E-04 | 1.82E-02 |
| **TMEM198B** | 1.418747452 | Upregulated | 1.09E-04 | 1.82E-02 |
| **ING2** | 1.822173407 | Upregulated | 1.11E-04 | 1.84E-02 |
| **PCBP1** | 1.35088942 | Upregulated | 1.16E-04 | 1.90E-02 |
| **HUWE1** | 1.496370086 | Upregulated | 1.17E-04 | 1.90E-02 |
| **LOC107986805** | 0.542248204 | Downregulated | 1.19E-04 | 1.92E-02 |
| **TLR1** | 0.457586886 | Downregulated | 1.20E-04 | 1.92E-02 |
| **CREB5** | 0.476242969 | Downregulated | 1.21E-04 | 1.92E-02 |
| **HCK** | 0.521368189 | Downregulated | 1.22E-04 | 1.92E-02 |
| **ADGRE3** | 0.420613085 | Downregulated | 1.22E-04 | 1.92E-02 |
| **PCMTD2** | 1.486372129 | Upregulated | 1.25E-04 | 1.94E-02 |
| **PISD** | 0.643675737 | Downregulated | 1.26E-04 | 1.94E-02 |
| **CEP19** | 0.562233735 | Downregulated | 1.35E-04 | 2.01E-02 |
| **RNF144B** | 0.550170441 | Downregulated | 1.35E-04 | 2.01E-02 |
| **NLRP12** | 0.5213452 | Downregulated | 1.36E-04 | 2.01E-02 |
| **CASP4** | 0.59117987 | Downregulated | 1.36E-04 | 2.01E-02 |
| **HYOU1** | 1.420634593 | Upregulated | 1.37E-04 | 2.01E-02 |
| **RAB33B** | 0.66902076 | Downregulated | 1.38E-04 | 2.01E-02 |
| **PNO1** | 1.511360224 | Upregulated | 1.38E-04 | 2.01E-02 |
| **PTAFR** | 0.687802213 | Downregulated | 1.38E-04 | 2.01E-02 |
| **MPPE1** | 0.66313569 | Downregulated | 1.40E-04 | 2.01E-02 |
| **TAF6** | 1.377443905 | Upregulated | 1.45E-04 | 2.06E-02 |
| **CD300A** | 0.587156545 | Downregulated | 1.46E-04 | 2.06E-02 |
| **FPR2** | 0.443316482 | Downregulated | 1.49E-04 | 2.08E-02 |
| **THRAP3** | 1.358068023 | Upregulated | 1.49E-04 | 2.08E-02 |
| **BAZ2B** | 0.550078362 | Downregulated | 1.56E-04 | 2.16E-02 |
| **BCL6** | 0.488522532 | Downregulated | 1.57E-04 | 2.16E-02 |
| **P2RY13** | 0.445690195 | Downregulated | 1.59E-04 | 2.17E-02 |
| **RAF1** | 0.641043923 | Downregulated | 1.59E-04 | 2.17E-02 |
| **NUMB** | 0.570367674 | Downregulated | 1.61E-04 | 2.17E-02 |
| **LST1** | 0.537433308 | Downregulated | 1.63E-04 | 2.17E-02 |
| **TLR5** | 0.511625887 | Downregulated | 1.63E-04 | 2.17E-02 |
| **PIH1D1** | 1.617988405 | Upregulated | 1.64E-04 | 2.17E-02 |
| **DENND3** | 0.519369846 | Downregulated | 1.65E-04 | 2.17E-02 |
| **MNDA** | 0.514258298 | Downregulated | 1.70E-04 | 2.22E-02 |
| **SNX4** | 1.559918952 | Upregulated | 1.80E-04 | 2.33E-02 |
| **CPQ** | 0.474870225 | Downregulated | 1.81E-04 | 2.33E-02 |
| **JAML** | 0.539485988 | Downregulated | 1.83E-04 | 2.35E-02 |
| **FPR1** | 0.501566477 | Downregulated | 1.86E-04 | 2.38E-02 |
| **CYB5R4** | 0.570981929 | Downregulated | 1.87E-04 | 2.38E-02 |
| **HMGN1P36** | 0.671684809 | Downregulated | 1.93E-04 | 2.39E-02 |
| **HTATSF1** | 1.632076844 | Upregulated | 1.93E-04 | 2.39E-02 |
| **ELOVL6** | 2.256479575 | Upregulated | 1.96E-04 | 2.39E-02 |
| **MMP25** | 0.558677916 | Downregulated | 1.99E-04 | 2.39E-02 |
| **CDR2** | 1.358801678 | Upregulated | 1.99E-04 | 2.39E-02 |
| **PYGL** | 0.448784784 | Downregulated | 1.99E-04 | 2.39E-02 |
| **UFC1** | 1.366382553 | Upregulated | 2.00E-04 | 2.39E-02 |
| **LOC100101246** | 0.718414285 | Downregulated | 2.01E-04 | 2.39E-02 |
| **GALNT3** | 0.622305821 | Downregulated | 2.02E-04 | 2.39E-02 |
| **ANXA3** | 0.362668643 | Downregulated | 2.03E-04 | 2.39E-02 |
| **DCK** | 1.591090624 | Upregulated | 2.05E-04 | 2.39E-02 |
| **CCNJL** | 0.603592087 | Downregulated | 2.05E-04 | 2.39E-02 |
| **TECPR2** | 0.604427903 | Downregulated | 2.06E-04 | 2.39E-02 |
| **EDIL3** | 2.04975978 | Upregulated | 2.06E-04 | 2.39E-02 |
| **PDCL** | 1.547679989 | Upregulated | 2.07E-04 | 2.39E-02 |
| **PPRC1** | 1.340016473 | Upregulated | 2.09E-04 | 2.40E-02 |
| **ALKBH7** | 1.455167657 | Upregulated | 2.10E-04 | 2.40E-02 |
| **TBXAS1** | 0.540933114 | Downregulated | 2.11E-04 | 2.40E-02 |
| **CA2** | 0.345667315 | Downregulated | 2.14E-04 | 2.41E-02 |
| **C5AR1** | 0.506022992 | Downregulated | 2.14E-04 | 2.41E-02 |
| **RUNX2** | 0.629472744 | Downregulated | 2.21E-04 | 2.44E-02 |
| **GULP1** | 3.34088166 | Upregulated | 2.21E-04 | 2.44E-02 |
| **PRDX4** | 1.513295826 | Upregulated | 2.22E-04 | 2.44E-02 |
| **FAM204A** | 1.30109072 | Upregulated | 2.24E-04 | 2.45E-02 |
| **MYLIP** | 1.433235875 | Upregulated | 2.29E-04 | 2.49E-02 |
| **LPCAT2** | 0.520729509 | Downregulated | 2.30E-04 | 2.49E-02 |
| **EPM2A** | 2.068065021 | Upregulated | 2.32E-04 | 2.49E-02 |
| **PAK1** | 0.648491515 | Downregulated | 2.34E-04 | 2.49E-02 |
| **S100A8** | 0.716462616 | Downregulated | 2.34E-04 | 2.49E-02 |
| **FAM126B** | 0.550450406 | Downregulated | 2.37E-04 | 2.51E-02 |
| **DPCD** | 1.918949889 | Upregulated | 2.38E-04 | 2.51E-02 |
| **RBM47** | 0.568728408 | Downregulated | 2.40E-04 | 2.51E-02 |
| **BNIP3** | 3.464236863 | Upregulated | 2.44E-04 | 2.54E-02 |
| **RAP2A** | 1.643198084 | Upregulated | 2.44E-04 | 2.54E-02 |
| **UXT** | 1.347537803 | Upregulated | 2.45E-04 | 2.54E-02 |
| **ATG9A** | 1.957437445 | Upregulated | 2.50E-04 | 2.57E-02 |
| **LGALS3** | 0.594206991 | Downregulated | 2.55E-04 | 2.61E-02 |
| **NOTCH1** | 0.716906296 | Downregulated | 2.58E-04 | 2.63E-02 |
| **RNF13** | 0.678390684 | Downregulated | 2.63E-04 | 2.66E-02 |
| **VMP1** | 0.492489954 | Downregulated | 2.65E-04 | 2.66E-02 |
| **NAMPT** | 0.567188972 | Downregulated | 2.67E-04 | 2.66E-02 |
| **C3orf62** | 0.624027588 | Downregulated | 2.70E-04 | 2.66E-02 |
| **LTBR** | 0.727249888 | Downregulated | 2.71E-04 | 2.66E-02 |
| **ABCF1** | 1.336687177 | Upregulated | 2.74E-04 | 2.66E-02 |
| **B4GALT3** | 2.003496898 | Upregulated | 2.78E-04 | 2.66E-02 |
| **RFWD2** | 0.577544849 | Downregulated | 2.79E-04 | 2.66E-02 |
| **WDR83OS** | 1.516717409 | Upregulated | 2.79E-04 | 2.66E-02 |
| **XPO6** | 0.57874028 | Downregulated | 2.79E-04 | 2.66E-02 |
| **TYROBP** | 0.611703656 | Downregulated | 2.79E-04 | 2.66E-02 |
| **FGR** | 0.607065724 | Downregulated | 2.82E-04 | 2.66E-02 |
| **C9orf84** | 0.423141306 | Downregulated | 2.82E-04 | 2.66E-02 |
| **LINC00173** | 0.689100963 | Downregulated | 2.82E-04 | 2.66E-02 |
| **PRR13** | 0.487342237 | Downregulated | 2.83E-04 | 2.66E-02 |
| **SNORA33** | 1.485437588 | Upregulated | 2.84E-04 | 2.66E-02 |
| **BID** | 0.657024655 | Downregulated | 2.85E-04 | 2.66E-02 |
| **VNN3** | 0.433372485 | Downregulated | 2.87E-04 | 2.68E-02 |
| **ALOX5AP** | 0.553455269 | Downregulated | 2.91E-04 | 2.68E-02 |
| **GRSF1** | 1.309503468 | Upregulated | 2.92E-04 | 2.68E-02 |
| **LAT2** | 0.750346315 | Downregulated | 2.94E-04 | 2.69E-02 |
| **TNFAIP6** | 0.406622025 | Downregulated | 3.00E-04 | 2.73E-02 |
| **RFXANK** | 1.50632974 | Upregulated | 3.02E-04 | 2.74E-02 |
| **POP7** | 1.65689854 | Upregulated | 3.08E-04 | 2.78E-02 |
| **ST6GALNAC2** | 0.520856573 | Downregulated | 3.10E-04 | 2.79E-02 |
| **BCL2L15** | 0.474687827 | Downregulated | 3.13E-04 | 2.80E-02 |
| **UBXN10** | 1.657779652 | Upregulated | 3.16E-04 | 2.80E-02 |
| **TRPV2** | 1.862980714 | Upregulated | 3.17E-04 | 2.80E-02 |
| **TMCC3** | 0.671438705 | Downregulated | 3.19E-04 | 2.80E-02 |
| **NOXRED1** | 0.728628039 | Downregulated | 3.22E-04 | 2.80E-02 |
| **LARP4** | 1.431193662 | Upregulated | 3.23E-04 | 2.80E-02 |
| **WLS** | 0.503459544 | Downregulated | 3.23E-04 | 2.80E-02 |
| **OSBPL1A** | 0.743393443 | Downregulated | 3.24E-04 | 2.80E-02 |
| **SKAP2** | 0.622298979 | Downregulated | 3.25E-04 | 2.80E-02 |
| **TOMM5** | 1.482020949 | Upregulated | 3.26E-04 | 2.80E-02 |
| **CAMP** | 0.285536367 | Downregulated | 3.26E-04 | 2.80E-02 |
| **CASP1** | 0.570409718 | Downregulated | 3.31E-04 | 2.81E-02 |
| **C14orf2** | 1.386013076 | Upregulated | 3.31E-04 | 2.81E-02 |
| **ZNF593** | 1.375627759 | Upregulated | 3.34E-04 | 2.81E-02 |
| **CEP295NL** | 0.665505789 | Downregulated | 3.35E-04 | 2.81E-02 |
| **NTNG2** | 0.71029968 | Downregulated | 3.35E-04 | 2.81E-02 |
| **GAB2** | 0.547573691 | Downregulated | 3.35E-04 | 2.81E-02 |
| **ALPK1** | 0.495958373 | Downregulated | 3.37E-04 | 2.81E-02 |
| **HSPA6** | 0.514416706 | Downregulated | 3.40E-04 | 2.82E-02 |
| **NCF4** | 0.523645732 | Downregulated | 3.45E-04 | 2.84E-02 |
| **MCM5** | 1.541910053 | Upregulated | 3.45E-04 | 2.84E-02 |
| **ALOX5** | 0.570837084 | Downregulated | 3.50E-04 | 2.87E-02 |
| **ABCA13** | 0.409488197 | Downregulated | 3.54E-04 | 2.88E-02 |
| **NABP1** | 0.492981233 | Downregulated | 3.54E-04 | 2.88E-02 |
| **TET2** | 0.574271548 | Downregulated | 3.56E-04 | 2.88E-02 |
| **RGS18** | 0.539966758 | Downregulated | 3.57E-04 | 2.88E-02 |
| **CFLAR** | 0.548396899 | Downregulated | 3.59E-04 | 2.88E-02 |
| **LIN7A** | 0.40816706 | Downregulated | 3.59E-04 | 2.88E-02 |
| **SERPINA1** | 0.571545136 | Downregulated | 3.60E-04 | 2.88E-02 |
| **ANKRD13C** | 1.407778074 | Upregulated | 3.65E-04 | 2.90E-02 |
| **PARP8** | 0.572601956 | Downregulated | 3.66E-04 | 2.90E-02 |
| **YIF1A** | 1.416004917 | Upregulated | 3.68E-04 | 2.90E-02 |
| **XKR8** | 0.763011383 | Downregulated | 3.71E-04 | 2.92E-02 |
| **SPATA1** | 0.514835934 | Downregulated | 3.74E-04 | 2.93E-02 |
| **FCGR2A** | 0.500372657 | Downregulated | 3.79E-04 | 2.95E-02 |
| **MCTP1** | 0.565560685 | Downregulated | 3.90E-04 | 3.02E-02 |
| **PTPRE** | 0.595833634 | Downregulated | 3.92E-04 | 3.02E-02 |
| **VAMP3** | 0.703791663 | Downregulated | 3.94E-04 | 3.02E-02 |
| **MAK** | 0.546780421 | Downregulated | 3.95E-04 | 3.02E-02 |
| **RGS2** | 0.407754338 | Downregulated | 3.95E-04 | 3.02E-02 |
| **ZNF285** | 1.431355769 | Upregulated | 3.97E-04 | 3.02E-02 |
| **MEA1** | 1.334806607 | Upregulated | 4.02E-04 | 3.04E-02 |
| **NUTF2P4** | 1.326631611 | Upregulated | 4.04E-04 | 3.05E-02 |
| **ST8SIA4** | 0.585797812 | Downregulated | 4.13E-04 | 3.08E-02 |
| **KIAA1551** | 0.637286361 | Downregulated | 4.14E-04 | 3.08E-02 |
| **ARHGAP26** | 0.545181676 | Downregulated | 4.15E-04 | 3.08E-02 |
| **CSF2RA** | 0.577216767 | Downregulated | 4.20E-04 | 3.10E-02 |
| **PDSS1P1** | 0.43353136 | Downregulated | 4.21E-04 | 3.10E-02 |
| **DOCK5** | 0.45396755 | Downregulated | 4.21E-04 | 3.10E-02 |
| **GADD45GIP1** | 1.541984583 | Upregulated | 4.26E-04 | 3.13E-02 |
| **RAB24** | 0.682082523 | Downregulated | 4.34E-04 | 3.15E-02 |
| **PSMC4** | 1.383383084 | Upregulated | 4.37E-04 | 3.15E-02 |
| **CEACAM4** | 0.587766551 | Downregulated | 4.37E-04 | 3.15E-02 |
| **SLU7** | 1.497907775 | Upregulated | 4.37E-04 | 3.15E-02 |
| **NME8** | 0.543344034 | Downregulated | 4.40E-04 | 3.16E-02 |
| **MX2** | 0.531692499 | Downregulated | 4.44E-04 | 3.16E-02 |
| **BRD4** | 1.339554222 | Upregulated | 4.45E-04 | 3.16E-02 |
| **CMTM1** | 0.551686537 | Downregulated | 4.47E-04 | 3.17E-02 |
| **MME** | 0.344236595 | Downregulated | 4.51E-04 | 3.18E-02 |
| **ZNF516** | 0.73969055 | Downregulated | 4.52E-04 | 3.18E-02 |
| **SNX10** | 0.585428681 | Downregulated | 4.56E-04 | 3.19E-02 |
| **SLC6A6** | 0.526204147 | Downregulated | 4.57E-04 | 3.19E-02 |
| **LIN54** | 1.352190004 | Upregulated | 4.59E-04 | 3.19E-02 |
| **NHSL2** | 0.546465109 | Downregulated | 4.61E-04 | 3.19E-02 |
| **AREL1** | 0.711393667 | Downregulated | 4.65E-04 | 3.19E-02 |
| **MYD88** | 0.696215173 | Downregulated | 4.66E-04 | 3.19E-02 |
| **NECAB2** | 0.718025195 | Downregulated | 4.68E-04 | 3.19E-02 |
| **DMXL2** | 0.54513348 | Downregulated | 4.68E-04 | 3.19E-02 |
| **RAB3D** | 0.59823348 | Downregulated | 4.70E-04 | 3.19E-02 |
| **LINC01126** | 0.718016159 | Downregulated | 4.70E-04 | 3.19E-02 |
| **SUGT1** | 1.383320386 | Upregulated | 4.71E-04 | 3.19E-02 |
| **MORC2** | 1.37318169 | Upregulated | 4.83E-04 | 3.24E-02 |
| **HSH2D** | 0.720425145 | Downregulated | 4.83E-04 | 3.24E-02 |
| **ZFAND5** | 1.509963266 | Upregulated | 4.84E-04 | 3.24E-02 |
| **STOML2** | 1.568636554 | Upregulated | 4.85E-04 | 3.24E-02 |
| **MSL1** | 0.636354495 | Downregulated | 4.86E-04 | 3.24E-02 |
| **VSIR** | 0.720433733 | Downregulated | 4.91E-04 | 3.26E-02 |
| **ZFYVE16** | 0.503736148 | Downregulated | 4.95E-04 | 3.28E-02 |
| **IFITM2** | 0.737578084 | Downregulated | 5.01E-04 | 3.31E-02 |
| **GNAI1** | 1.726047528 | Upregulated | 5.06E-04 | 3.33E-02 |
| **S100A12** | 0.443677371 | Downregulated | 5.08E-04 | 3.33E-02 |
| **ARF4** | 1.643781812 | Upregulated | 5.08E-04 | 3.33E-02 |
| **CLEC4D** | 0.410500167 | Downregulated | 5.16E-04 | 3.35E-02 |
| **DAPP1** | 0.56736244 | Downregulated | 5.17E-04 | 3.35E-02 |
| **CORO1C** | 1.478117074 | Upregulated | 5.18E-04 | 3.35E-02 |
| **SPI1** | 0.614464487 | Downregulated | 5.21E-04 | 3.35E-02 |
| **IGSF6** | 0.566044305 | Downregulated | 5.22E-04 | 3.35E-02 |
| **ANTXR2** | 0.63091053 | Downregulated | 5.22E-04 | 3.35E-02 |
| **TLR4** | 0.449860481 | Downregulated | 5.23E-04 | 3.35E-02 |
| **HRH2** | 0.524676857 | Downregulated | 5.26E-04 | 3.35E-02 |
| **RASSF2** | 0.561791332 | Downregulated | 5.33E-04 | 3.39E-02 |
| **HSP90AB1** | 1.534465736 | Upregulated | 5.39E-04 | 3.42E-02 |
| **MIR1247** | 1.525502907 | Upregulated | 5.42E-04 | 3.42E-02 |
| **GNL2** | 1.432011574 | Upregulated | 5.43E-04 | 3.42E-02 |
| **CELF2-AS1** | 0.543189438 | Downregulated | 5.47E-04 | 3.43E-02 |
| **APOBEC3A** | 0.531002231 | Downregulated | 5.52E-04 | 3.45E-02 |
| **FAM212B** | 0.684611108 | Downregulated | 5.52E-04 | 3.45E-02 |
| **CLEC4A** | 0.528464951 | Downregulated | 5.54E-04 | 3.45E-02 |
| **TNFRSF1A** | 0.701435325 | Downregulated | 5.57E-04 | 3.46E-02 |
| **THBS1** | 2.455185478 | Upregulated | 5.65E-04 | 3.49E-02 |
| **SSRP1** | 1.351828582 | Upregulated | 5.69E-04 | 3.49E-02 |
| **KY** | 0.616375056 | Downregulated | 5.69E-04 | 3.49E-02 |
| **TLR2** | 0.495757481 | Downregulated | 5.71E-04 | 3.49E-02 |
| **FKBP15** | 0.735402458 | Downregulated | 5.74E-04 | 3.49E-02 |
| **FCAR** | 0.527193162 | Downregulated | 5.77E-04 | 3.50E-02 |
| **CXCR4** | 0.665039168 | Downregulated | 5.92E-04 | 3.58E-02 |
| **MSRB1** | 0.576225657 | Downregulated | 5.96E-04 | 3.59E-02 |
| **RUVBL1** | 1.469140653 | Upregulated | 6.01E-04 | 3.61E-02 |
| **CCT2** | 1.586624429 | Upregulated | 6.05E-04 | 3.63E-02 |
| **FRY** | 0.577972684 | Downregulated | 6.12E-04 | 3.66E-02 |
| **PHC2** | 0.734418787 | Downregulated | 6.21E-04 | 3.70E-02 |
| **KPNA4** | 1.32865022 | Upregulated | 6.31E-04 | 3.75E-02 |
| **NAA50** | 1.317177326 | Upregulated | 6.35E-04 | 3.75E-02 |
| **HAL** | 0.487442507 | Downregulated | 6.35E-04 | 3.75E-02 |
| **IMPDH2** | 1.39045609 | Upregulated | 6.43E-04 | 3.79E-02 |
| **MBOAT7** | 0.653306157 | Downregulated | 6.45E-04 | 3.79E-02 |
| **KDELR2** | 1.455963606 | Upregulated | 6.51E-04 | 3.82E-02 |
| **ARNTL** | 0.612710155 | Downregulated | 6.66E-04 | 3.87E-02 |
| **CLEC7A** | 0.435836588 | Downregulated | 6.67E-04 | 3.87E-02 |
| **CCR3** | 0.649133193 | Downregulated | 6.67E-04 | 3.87E-02 |
| **LRRK1** | 0.759789492 | Downregulated | 6.69E-04 | 3.88E-02 |
| **STX3** | 0.507674816 | Downregulated | 6.79E-04 | 3.92E-02 |
| **IRAK3** | 0.493508885 | Downregulated | 6.80E-04 | 3.92E-02 |
| **SMCHD1** | 0.614486577 | Downregulated | 6.83E-04 | 3.92E-02 |
| **CMTM2** | 0.610967707 | Downregulated | 6.85E-04 | 3.92E-02 |
| **ELOVL5** | 0.68104462 | Downregulated | 6.88E-04 | 3.93E-02 |
| **TSEN34** | 0.72966718 | Downregulated | 6.91E-04 | 3.93E-02 |
| **CLUAP1** | 1.399218111 | Upregulated | 6.91E-04 | 3.93E-02 |
| **DUSP6** | 0.681857795 | Downregulated | 7.02E-04 | 3.95E-02 |
| **ATP5J** | 1.343762826 | Upregulated | 7.04E-04 | 3.95E-02 |
| **HSPD1** | 1.425121376 | Upregulated | 7.05E-04 | 3.95E-02 |
| **MIRLET7I** | 0.730195441 | Downregulated | 7.07E-04 | 3.95E-02 |
| **NFAM1** | 0.577761793 | Downregulated | 7.07E-04 | 3.95E-02 |
| **ALDH1A1** | 0.681566867 | Downregulated | 7.09E-04 | 3.95E-02 |
| **CCDC167** | 1.489867179 | Upregulated | 7.10E-04 | 3.95E-02 |
| **PRDX1** | 1.508363539 | Upregulated | 7.21E-04 | 3.98E-02 |
| **C15orf39** | 0.693230635 | Downregulated | 7.22E-04 | 3.98E-02 |
| **BNIP3P1** | 1.352771279 | Upregulated | 7.24E-04 | 3.98E-02 |
| **PLBD2** | 1.572929654 | Upregulated | 7.26E-04 | 3.98E-02 |
| **ASNA1** | 1.556264778 | Upregulated | 7.29E-04 | 3.98E-02 |
| **SRP72** | 1.33994245 | Upregulated | 7.32E-04 | 3.98E-02 |
| **PLAUR** | 0.575245083 | Downregulated | 7.32E-04 | 3.98E-02 |
| **EIF2S1** | 1.365655273 | Upregulated | 7.38E-04 | 4.01E-02 |
| **VNN2** | 0.496583643 | Downregulated | 7.43E-04 | 4.02E-02 |
| **ARSG** | 0.69966159 | Downregulated | 7.50E-04 | 4.03E-02 |
| **MICALCL** | 1.775608424 | Upregulated | 7.52E-04 | 4.03E-02 |
| **RASGRP4** | 0.599976034 | Downregulated | 7.55E-04 | 4.03E-02 |
| **C14orf159** | 0.640403327 | Downregulated | 7.55E-04 | 4.03E-02 |
| **SLPI** | 0.34427469 | Downregulated | 7.55E-04 | 4.03E-02 |
| **P2RY14** | 0.539265512 | Downregulated | 7.63E-04 | 4.06E-02 |
| **PHF23** | 1.32196037 | Upregulated | 7.68E-04 | 4.07E-02 |
| **IFNK** | 0.630421555 | Downregulated | 7.70E-04 | 4.07E-02 |
| **KAT14** | 1.31890508 | Upregulated | 7.71E-04 | 4.07E-02 |
| **MGAM** | 0.449722129 | Downregulated | 7.79E-04 | 4.08E-02 |
| **NIN** | 0.674465536 | Downregulated | 7.83E-04 | 4.08E-02 |
| **HDLBP** | 1.356333501 | Upregulated | 7.83E-04 | 4.08E-02 |
| **GPR141** | 0.449622646 | Downregulated | 7.84E-04 | 4.08E-02 |
| **ZNF467** | 0.70863077 | Downregulated | 7.85E-04 | 4.08E-02 |
| **ROPN1L** | 0.537228626 | Downregulated | 7.86E-04 | 4.08E-02 |
| **SLC11A1** | 0.536876797 | Downregulated | 7.91E-04 | 4.08E-02 |
| **SLC45A4** | 0.642586685 | Downregulated | 7.93E-04 | 4.08E-02 |
| **MCM2** | 1.33540145 | Upregulated | 8.00E-04 | 4.09E-02 |
| **HSD17B11** | 0.660981096 | Downregulated | 8.00E-04 | 4.09E-02 |
| **LRRK2** | 0.456298009 | Downregulated | 8.02E-04 | 4.09E-02 |
| **TCN1** | 0.28863638 | Downregulated | 8.15E-04 | 4.15E-02 |
| **CDA** | 0.468917574 | Downregulated | 8.23E-04 | 4.18E-02 |
| **APAF1** | 0.608225365 | Downregulated | 8.27E-04 | 4.18E-02 |
| **EPB41L4A-AS1** | 1.357842088 | Upregulated | 8.28E-04 | 4.18E-02 |
| **NARS** | 1.426131899 | Upregulated | 8.32E-04 | 4.19E-02 |
| **LPGAT1** | 0.619725221 | Downregulated | 8.32E-04 | 4.19E-02 |
| **DPAGT1** | 1.334002369 | Upregulated | 8.34E-04 | 4.19E-02 |
| **ATF5** | 1.429168325 | Upregulated | 8.41E-04 | 4.20E-02 |
| **ATIC** | 1.559717536 | Upregulated | 8.41E-04 | 4.20E-02 |
| **PPP4R1** | 0.575170994 | Downregulated | 8.43E-04 | 4.20E-02 |
| **ARL11** | 0.610169484 | Downregulated | 8.50E-04 | 4.21E-02 |
| **PPCDC** | 0.715694601 | Downregulated | 8.66E-04 | 4.26E-02 |
| **EMG1** | 1.391523202 | Upregulated | 8.67E-04 | 4.26E-02 |
| **ELP6** | 1.307880571 | Upregulated | 8.68E-04 | 4.26E-02 |
| **RAN** | 1.501831146 | Upregulated | 8.73E-04 | 4.28E-02 |
| **AHI1** | 1.362203165 | Upregulated | 8.90E-04 | 4.35E-02 |
| **ZNF410** | 1.403026935 | Upregulated | 8.95E-04 | 4.36E-02 |
| **WDFY3** | 0.477968028 | Downregulated | 9.01E-04 | 4.38E-02 |
| **LYZ** | 0.7143427 | Downregulated | 9.03E-04 | 4.38E-02 |
| **DCUN1D5** | 1.340891988 | Upregulated | 9.06E-04 | 4.39E-02 |
| **TSN** | 1.367701719 | Upregulated | 9.11E-04 | 4.40E-02 |
| **GTF3C6** | 1.433593092 | Upregulated | 9.18E-04 | 4.41E-02 |
| **JAK2** | 0.652698306 | Downregulated | 9.18E-04 | 4.41E-02 |
| **CCDC91** | 1.466570973 | Upregulated | 9.22E-04 | 4.41E-02 |
| **RNF175** | 0.584044168 | Downregulated | 9.22E-04 | 4.41E-02 |
| **CNEP1R1** | 0.703653036 | Downregulated | 9.26E-04 | 4.41E-02 |
| **HIPK1** | 1.314217893 | Upregulated | 9.30E-04 | 4.42E-02 |
| **LMAN1** | 1.496769738 | Upregulated | 9.37E-04 | 4.44E-02 |
| **PLB1** | 0.640565174 | Downregulated | 9.40E-04 | 4.44E-02 |
| **MYO1F** | 0.594612975 | Downregulated | 9.41E-04 | 4.44E-02 |
| **SLA** | 0.637051513 | Downregulated | 9.49E-04 | 4.47E-02 |
| **FUT8** | 1.394493938 | Upregulated | 9.63E-04 | 4.50E-02 |
| **GOLPH3L** | 1.495838696 | Upregulated | 9.69E-04 | 4.50E-02 |
| **STEAP4** | 0.471391129 | Downregulated | 9.72E-04 | 4.50E-02 |
| **CDK2** | 1.36879306 | Upregulated | 9.72E-04 | 4.50E-02 |
| **TNFRSF1B** | 0.700803271 | Downregulated | 9.73E-04 | 4.50E-02 |
| **ARPC5** | 0.741710819 | Downregulated | 9.73E-04 | 4.50E-02 |
| **TXNL4A** | 1.401943583 | Upregulated | 9.79E-04 | 4.52E-02 |
| **MARCKS** | 0.7417755 | Downregulated | 9.85E-04 | 4.53E-02 |
| **ATP5I** | 1.459557047 | Upregulated | 9.91E-04 | 4.55E-02 |
| **IGF2BP2** | 1.632306489 | Upregulated | 9.95E-04 | 4.56E-02 |
| **GPAT2P2** | 0.731879624 | Downregulated | 9.97E-04 | 4.56E-02 |
| **FGD4** | 0.604347272 | Downregulated | 1.01E-03 | 4.58E-02 |
| **CDC42EP3** | 0.662764653 | Downregulated | 1.01E-03 | 4.58E-02 |
| **AMN1** | 0.654546635 | Downregulated | 1.01E-03 | 4.58E-02 |
| **SAMM50** | 1.328554074 | Upregulated | 1.02E-03 | 4.60E-02 |
| **DPYD** | 0.619434026 | Downregulated | 1.03E-03 | 4.63E-02 |
| **SVIL** | 0.662591091 | Downregulated | 1.03E-03 | 4.63E-02 |
| **MLKL** | 0.59442444 | Downregulated | 1.04E-03 | 4.63E-02 |
| **SERPINI1** | 2.634578166 | Upregulated | 1.04E-03 | 4.63E-02 |
| **STX11** | 0.611763811 | Downregulated | 1.04E-03 | 4.63E-02 |
| **TRPM6** | 0.528985534 | Downregulated | 1.05E-03 | 4.66E-02 |
| **SLC39A8** | 1.459876188 | Upregulated | 1.05E-03 | 4.66E-02 |
| **RALB** | 0.543720481 | Downregulated | 1.06E-03 | 4.68E-02 |
| **UBFD1** | 1.370536487 | Upregulated | 1.06E-03 | 4.68E-02 |
| **ASB7** | 1.421323392 | Upregulated | 1.07E-03 | 4.70E-02 |
| **CCNG2** | 0.692193539 | Downregulated | 1.08E-03 | 4.73E-02 |
| **RPS15A** | 1.63004989 | Upregulated | 1.08E-03 | 4.73E-02 |
| **HDAC2** | 1.335472147 | Upregulated | 1.09E-03 | 4.73E-02 |
| **PSEN1** | 0.723081663 | Downregulated | 1.09E-03 | 4.73E-02 |
| **KLHDC3** | 1.304486411 | Upregulated | 1.09E-03 | 4.73E-02 |
| **MRPS16** | 1.32598667 | Upregulated | 1.09E-03 | 4.73E-02 |
| **1-Mar** | 0.565549403 | Downregulated | 1.09E-03 | 4.73E-02 |
| **PIK3R1** | 1.320579707 | Upregulated | 1.10E-03 | 4.73E-02 |
| **SMDT1** | 1.399284422 | Upregulated | 1.10E-03 | 4.75E-02 |
| **AP5B1** | 0.6616479 | Downregulated | 1.10E-03 | 4.75E-02 |
| **CKS2** | 1.786661849 | Upregulated | 1.11E-03 | 4.78E-02 |
| **TUBA1A** | 0.688674663 | Downregulated | 1.12E-03 | 4.79E-02 |
| **CLEC4E** | 0.46764564 | Downregulated | 1.12E-03 | 4.79E-02 |
| **HCFC1** | 1.576247406 | Upregulated | 1.12E-03 | 4.79E-02 |
| **SIRPB1** | 0.52960974 | Downregulated | 1.13E-03 | 4.79E-02 |
| **ZRANB1** | 1.443089906 | Upregulated | 1.14E-03 | 4.83E-02 |
| **ZNF777** | 1.361157145 | Upregulated | 1.14E-03 | 4.83E-02 |
| **BMS1P20** | 1.847890244 | Upregulated | 1.15E-03 | 4.83E-02 |
| **PREX1** | 0.694233004 | Downregulated | 1.16E-03 | 4.87E-02 |
| **PRDM5** | 0.714625594 | Downregulated | 1.16E-03 | 4.87E-02 |
| **STRAP** | 1.518075612 | Upregulated | 1.17E-03 | 4.87E-02 |
| **NAA10** | 1.313806429 | Upregulated | 1.17E-03 | 4.87E-02 |
| **NLRP6** | 0.732054775 | Downregulated | 1.17E-03 | 4.88E-02 |
| **HCAR2** | 0.431162184 | Downregulated | 1.18E-03 | 4.89E-02 |
| **CPPED1** | 0.485909121 | Downregulated | 1.19E-03 | 4.92E-02 |
| **NCL** | 1.3391862 | Upregulated | 1.20E-03 | 4.94E-02 |
| **COPS2** | 1.512671045 | Upregulated | 1.20E-03 | 4.96E-02 |
| **DENND5A** | 0.698653384 | Downregulated | 1.22E-03 | 4.99E-02 |
| **AGO4** | 0.550445223 | Downregulated | 1.22E-03 | 5.00E-02 |
| **TNFSF14** | 0.727283947 | Downregulated | 1.22E-03 | 5.00E-02 |
| **ABHD12B** | 0.505014369 | Downregulated | 1.23E-03 | 5.00E-02 |

**Suppl. Table 5. Significant genes from cord blood analysis.** FC_GSE73685, fold-change calculated using GSE73685 samples; adj.P.Val, adjusted p-value.
