# Supplementary material for: Meta-Analysis of Maternal and Fetal Transcriptomic Data Elucidates the Role of Adaptive and Innate Immunity in Preterm Birth
Source: Front Immunol. 2018 May 9;9:993. doi: 10.3389/fimmu.2018.00993 (PMC5954243; doi:10.3389/fimmu.2018.00993)
Supplement: Supplementary file 6 [file Image_1.PDF]

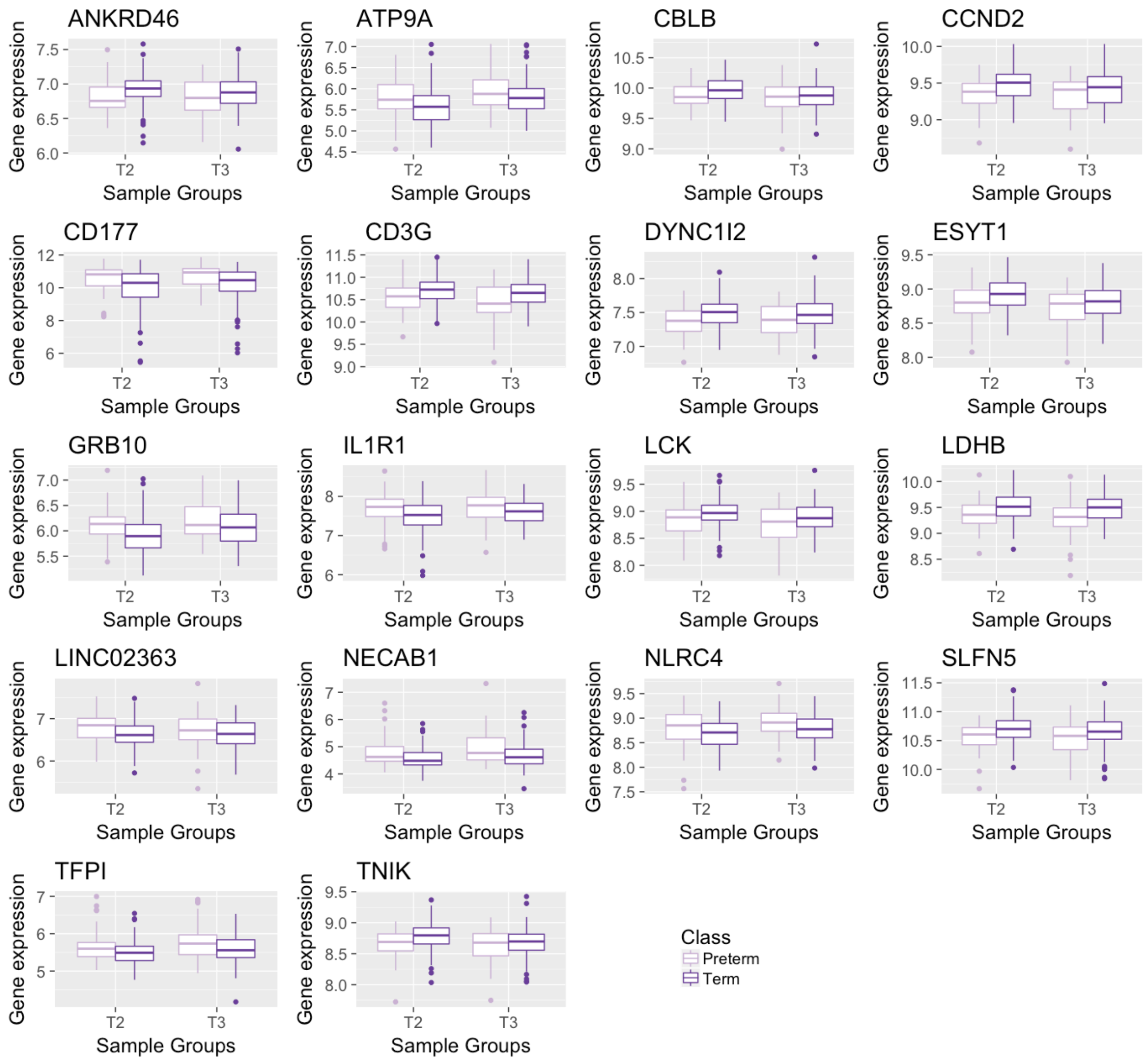

Suppl. Figure 1. Boxplots of raw gene expression values of overlapping significant genes from T2 analysis and meta-analysis. Raw gene expression from GSE59491 plotted.
